# Systems Modeling of Molecular Mechanisms Controlling Cytokine-driven CD4+ T Cell Differentiation and Phenotype Plasticity

**DOI:** 10.1371/journal.pcbi.1003027

**Published:** 2013-04-04

**Authors:** Adria Carbo, Raquel Hontecillas, Barbara Kronsteiner, Monica Viladomiu, Mireia Pedragosa, Pinyi Lu, Casandra W. Philipson, Stefan Hoops, Madhav Marathe, Stephen Eubank, Keith Bisset, Katherine Wendelsdorf, Abdul Jarrah, Yongguo Mei, Josep Bassaganya-Riera

**Affiliations:** 1 Nutritional Immunology and Molecular Medicine Laboratory, Virginia Bioinformatics Institute, Virginia Tech, Blacksburg, Virginia, United States of America; 2 Center for Modeling Immunity to Enteric Pathogens, Virginia Bioinformatics Institute, Virginia Tech, Blacksburg, Virginia, United States of America; 3 Network Dynamics and Simulation Science Laboratory, Virginia Bioinformatics Institute, Virginia Tech, Blacksburg, Virginia, United States of America; 4 Department of Biomedical Sciences and Pathobiology, Virginia-Maryland Regional College of Veterinary Medicine, Virginia Tech, Blacksburg, Virginia, United States of America; Johns Hopkins University, United States of America

## Abstract

Differentiation of CD4+ T cells into effector or regulatory phenotypes is tightly controlled by the cytokine milieu, complex intracellular signaling networks and numerous transcriptional regulators. We combined experimental approaches and computational modeling to investigate the mechanisms controlling differentiation and plasticity of CD4+ T cells in the gut of mice. Our computational model encompasses the major intracellular pathways involved in CD4+ T cell differentiation into T helper 1 (Th1), Th2, Th17 and induced regulatory T cells (iTreg). Our modeling efforts predicted a critical role for peroxisome proliferator-activated receptor gamma (PPARγ) in modulating plasticity between Th17 and iTreg cells. PPARγ regulates differentiation, activation and cytokine production, thereby controlling the induction of effector and regulatory responses, and is a promising therapeutic target for dysregulated immune responses and inflammation. Our modeling efforts predict that following PPARγ activation, Th17 cells undergo phenotype switch and become iTreg cells. This prediction was validated by results of adoptive transfer studies showing an increase of colonic iTreg and a decrease of Th17 cells in the gut mucosa of mice with colitis following pharmacological activation of PPARγ. Deletion of PPARγ in CD4+ T cells impaired mucosal iTreg and enhanced colitogenic Th17 responses in mice with CD4+ T cell-induced colitis. Thus, for the first time we provide novel molecular evidence *in vivo* demonstrating that PPARγ in addition to regulating CD4+ T cell differentiation also plays a major role controlling Th17 and iTreg plasticity in the gut mucosa.

## Author Summary

CD4+ T cells can differentiate into different phenotypes depending on the cytokine milieu. Due to the complexity of this process, we have constructed a computational and mathematical model with sixty ordinary differential equations representing a CD4+ T cell differentiating into either Th1, Th2, Th17 or iTreg cells. The model includes cytokines, nuclear receptors and transcription factors that define fate and function of CD4+ T cells. Computational simulations illustrate how a proinflammatory Th17 cell can undergo reprogramming into an anti-inflammatory iTreg phenotype following PPARγ activation. This modeling-derived hypothesis has been validated with *in vitro* and *in vivo* experiments. Experimental data support the modeling-derived prediction and demonstrate that the loss of PPARγ enhances a proinflammatory response characterized by Th17 in colitis-induced mice. Moreover, pharmacological activation of PPARγ *in vivo* can affect the Th17/iTreg balance by upregulating FOXP3 and downregulating IL-17A and RORγt. In summary, we demonstrate that computational simulations using our CD4+ T cell model provide novel unforeseen hypotheses related to the molecular mechanisms controlling differentiation and function of CD4+ T cells. *In vivo* findings validated the modeling prediction that PPARγ modulates differentiation and plasticity of CD4+ T cells in mice.

## Introduction

The CD4+ T cell differentiation process activates the transcriptional and secretory cellular machinery that helps orchestrate immune modulation in infectious, allergic and immune-mediated diseases. Upon antigen presentation, naïve CD4+ T cells become activated and undergo a differentiation process controlled by the cytokine milieu in the tissue environment. For instance, interleukin-6 (IL-6) in combination with transforming growing factor β (TGF-β) trigger a naive CD4+ T cell to become a T helper 17 (Th17) cell [1], [2]. In contrast, TGF-β alone can activate regulatory pathways leading to differentiation of naive CD4+ T cells into an induced regulatory CD4+ T cell (iTreg) phenotype, which in turn tightly dampens effector and inflammatory responses.

CD4+ T cell differentiation was once viewed as a rigid process whereby a naive cell differentiated into terminal phenotypes. However, mounting evidence supports the tissue environment-dependent plasticity of CD4+ T cell subsets and suggests the emergence of new phenotypes [3]–[5]. At the molecular level, plasticity is achieved by a cytokine-driven reprogramming of signaling pathways and targeted activation of master regulator transcription factors which results in gene expression changes [6]. Antigen presenting cells (APCs) influence T cell differentiation through antigen presentation, co-stimulation and cytokine secretion [7]. The crosstalk between T cell phenotypes has been fully characterized in terms of classical Th1 versus Th2 differentiation [8]–[11]. Indeed, a logical network model of CD4+ T cell differentiation process centered around Th1 versus Th2 differentiation was published by Mendoza [12]. However, this logical model did not consider the Th17 or iTreg cell subsets. In the last decade, Th17 has emerged as an extremely plastic phenotype [6], [13]–[15] that can acquire regulatory functions following changes in the local cytokine milieu [16]–[19]. Furthermore, human iTreg cells become interleukin-17 (IL-17)-producing Th17 cells [20], thereby supporting the concept that Th17 plasticity is a two-way process. However, the molecular mechanisms underlying these processes are incompletely understood.

Retinoic acid receptor-related orphan receptor gamma (RORγt) is a master regulator transcription factor required for Th17 differentiation [21], [22] and it has been proposed as a potential therapeutic target to suppress Th17 responses in autoimmune diseases [23], [24]. Similar to RORγt, the peroxisome proliferator-activated receptors (PPARs) are ligand-activated transcription factors and members of the nuclear receptor superfamily. PPARγ is highly expressed in CD4+ T cells and it has been reported to modulate Th1 and natural Treg (nTreg) function [25]–[27], but limited information is available regarding its role in modulating the Th17 and iTreg phenotypes. The loss of PPARγ in CD4+ T cells enhanced antigen-specific proliferation and overproduction of interferon γ (IFN-γ) in response to IL-12 [28]. In addition, the deficiency of PPARγ in nTreg cells impairs their ability to prevent effector T cell-induced colitis following transfer of naïve CD4+ T cells into SCID recipients [28]. Furthermore, pharmacologic activation of PPARγ prevents removal of the silencing mediator for retinoid and thyroid hormone receptors' co-repressor from the RORγt promoter in T cells, thus interfering with RORγt transcription [29]. While previous studies shed some light on the role of PPARγ in Th17 differentiation, this is the first study to investigate the role of PPARγ in controlling Th17 to iTreg cell plasticity in the gut mucosa.

Computational approaches have become a powerful tool that allows concurrent multiparametric analysis of dynamic biological processes and diseases. The emerging use of systems modeling in combination with experimental immunology studies *in vivo* can help integrate existing knowledge and provide novel insights on rising trends and behaviors in biological processes such as CD4+ T cell differentiation and function. Of note, bioengineering studies demonstrated the predictive value of a whole-cell computational model of the life cycle of *Mycoplasma genitalium* [30]. These multi-mode calibrated models demonstrate an emerging strategy to answer questions about fundamental cell-based processes *in silico* and help focus experimental designs of animal pre-clinical and human clinical studies.

We combined computational modeling and mouse adoptive transfer studies to gain a better mechanistic understanding of the modulation of CD4+ T cell differentiation and plasticity at the intestinal mucosa of mice. Our sensitivity analyses highlighted the importance of PPARγ in the regulation of Th17 to iTreg plasticity. Indeed, *in vivo* evidence demonstrates that PPARγ is required for the plasticity of Th17 promoting a functional shift towards an iTreg phenotype. More specifically, PPARγ activation is associated with upregulation of FOXP3 and suppression IL-17A and RORγt expression in colonic lamina propria CD4+ T cells. Conversely, the loss of PPARγ in T cells results in colonic immunopathology driven by Th17 cells in adoptive transfer studies.

## Results

### Mathematical modeling of intracellular cytokine pathways controlling CD4+ T cell differentiation

**Figure 1:**
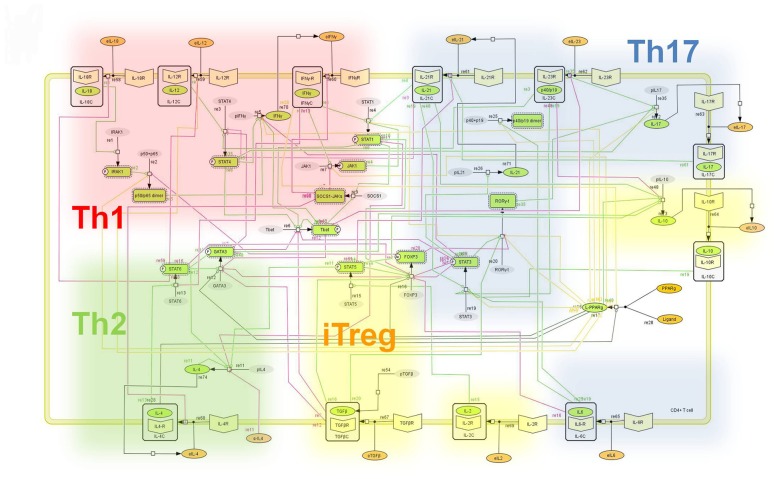
Network model illustrating the complex intracellular signaling pathways and transcriptional factors controlling the CD4+ T cell differentiation process. The signaling network illustrates network topologies associated with differentiation towards T helper (Th)1 (red shadow), Th2 (green shadow), Th17 (blue shadow) and induced regulatory T cells (iTreg, yellow shadow). The network is provided in Systems Biology Markup Language-compliant format.

Cytokines are small molecules secreted in response to external stimuli, which are key in cell-to-cell communication. Cytokine signaling is fast and canonical, consisting of 1) binding to cytokine cell surface receptor, 2) activation of receptor-associated kinase, 3) STAT phosphorylation and translocation into the nucleus and 4) activation of gene expression. In naïve CD4+ T cells cytokine signaling leads to the expression of transcription factors that upregulate gene subsets that shape cell phenotype and function. As an output of this process, differentiated cells preferentially secrete phenotype–associated cytokines, such as IL-17 produced by Th17 cells or IFNy produced by Th1 (Figure S1). To facilitate a comprehensive representation of the dynamics associated with the major pathways activated by cytokines which control CD4+ T cell differentiation and plasticity, we constructed an ordinary differential equation (ODE)-based computational model including cytokines, membrane receptors and transcription factors (Figure 1). Knowledge discovery involved an iterative process that fully integrated computational modeling and *in vivo* experimentation in mice (Figure S2). The CD4+ T cell differentiation model consists of 60 ODEs, 52 reactions and 93 species (Figure S3). The mathematical model was engineered to ensure proper modulation of intracellular pathways and cell phenotypes via external cytokines representing the cytokine milieu. The Hill Function and mass action equations were used [31]. While the Hill Coefficient allowed us to quantify the effect of a ligand binding a macromolecule through cooperative binding, mass action laws can represent dynamic equilibriums for elementary reactions, considering products as a proportion of the participating molecules in the reaction. Experimental data (Table S1) was used to calibrate and adjust model parameters to ensure correct dynamics (Table S2 and S3, Figure S4). A list of modeling assumptions can be found in Table S4. Among the four possible phenotypes in this mathematical model, to induce Th17 differentiation from a naïve CD4+ T cell, external IL-6 and external TGF-β were added in combination and demonstrated upregulation of RORγt, IL-17 and STAT-3 (Figure S5) as followed by our table of initialization fates (Table S5). Sensitivity analyses identified PPARγ as an essential regulator of CD4+ T cell differentiation and plasticity (Figure S6).

### PPARγ plays an essential role in modulating CD4+ T cell differentiation and plasticity in a dose-dependent manner

**Figure 2:**
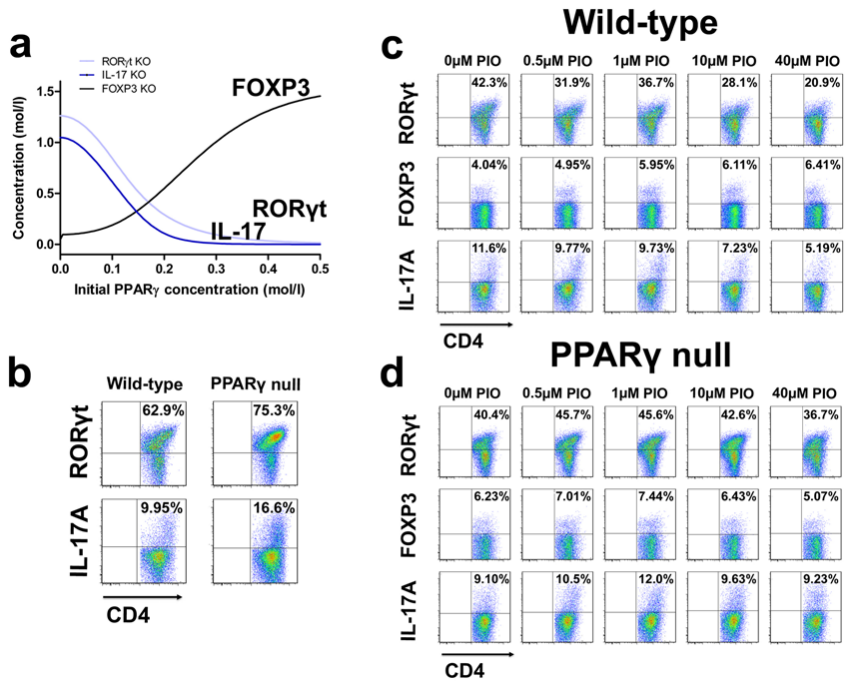
Activation of peroxisome proliferator-activated receptor γ (PPARγ) regulates differentiation of CD4+ T cells. (A) Computational simulation of the effect of *in silico* activation of PPARγ in a T helper (Th)17 cell on the levels of FOXP3, IL-17 and RORγt. (B) PPARγ inhibits Th17 differentiation. Naïve wild-type CD4+ T cells differentiated with IL-6 in combination with TGF-β *in vitro* for 60h express less RORγt and produce lower levels of IL-17A when compared to T cell-specific PPARγ null Th17 cells. (C) Increasing concentrations of pioglitazone (PIO), a full PPARγ agonist, upregulate FOXP3 in wild-type Th17 differentiated cells following 24 h treatment and down-regulate RORγt and IL-17A in wild-type cells. (D) Increasing concentrations of PIO do not have an effect in PPARγ null Th17 cells. The double-positive region can be observed in the upper right part of the flow plots.

Based on the results of the sensitivity analysis we performed computer simulations aimed to further characterize the role of PPARγ on Th cell differentiation *in silico*. Following induction of the computational model towards a Th17 phenotype by adding external TGF-β and external IL-6 *in silico*, modeling efforts predicted that increasing concentrations of PPARγ in Th17 cells led to downregulation of RORγt and IL-17 and upregulation of FOXP3 (Figure 2A), thus, displaying a phenotype switch from Th17 to iTreg. A list of computational modeling derived predictions can be found in Table S6. To validate the results of our computational simulations, we first isolated and sorted naïve CD4+ T cells from spleens of wild-type and T cell-specific PPARγ null mice. Deletion of PPARγ via a transgenic expression of Cre under control of the *CD4* promoter (PPARγ^fl/fl^; CD4-Cre+) allowed us to use loss-of-function approaches to characterize the role of PPARγ in Th17 differentiation. Cells were polarized towards a Th17 phenotype with recombinant mouse IL-6 and TGF-β. IFNγ and IL-4 were eliminated to block Th1 and Th2 differentiation respectively with neutralizing antibodies. After 60 hours of culture, cells were treated with increasing amounts of pioglitazone (PIO), a synthetic PPARγ agonist of the thiazolidinedione (TZD) class of anti-diabetic drugs. Before starting pioglitazone treatment, at t = 60 h, IL-17 and RORγt expression were significantly upregulated in PPARγ null when compared to wild-type cells (Figure 2B). Following pioglitazone treatment for 24 h., Th17 cells from wild-type mice showed increasing levels of FOXP3 and downregulation of RORγt and IL-17A with increased concentration of the exogenous PPARγ agonist in wild-type (Figure 2C), but this effect was not observed in PPARγ null Th17 cells (Figure 2D), suggesting the role of PPARγ in the modulation of these molecules. The same study was repeated three times with very similar trends on these behaviors (Figure S7). These results provide *in vitro* evidence that PPARγ significantly dampens Th17 differentiation and slightly enhances FOXP3 expression. Interestingly, uncoupling between suppressed Th17 responses and enhanced iTreg cells suggests that a T cell-extrinsic mechanism (i.e., APC-derived signals) might be contributing to this Th17 plasticity *in vivo*.

### The lack of PPARγ in naïve CD4+ T cells impairs their ability to differentiate into iTreg cells *in vivo*

**Figure 3:**
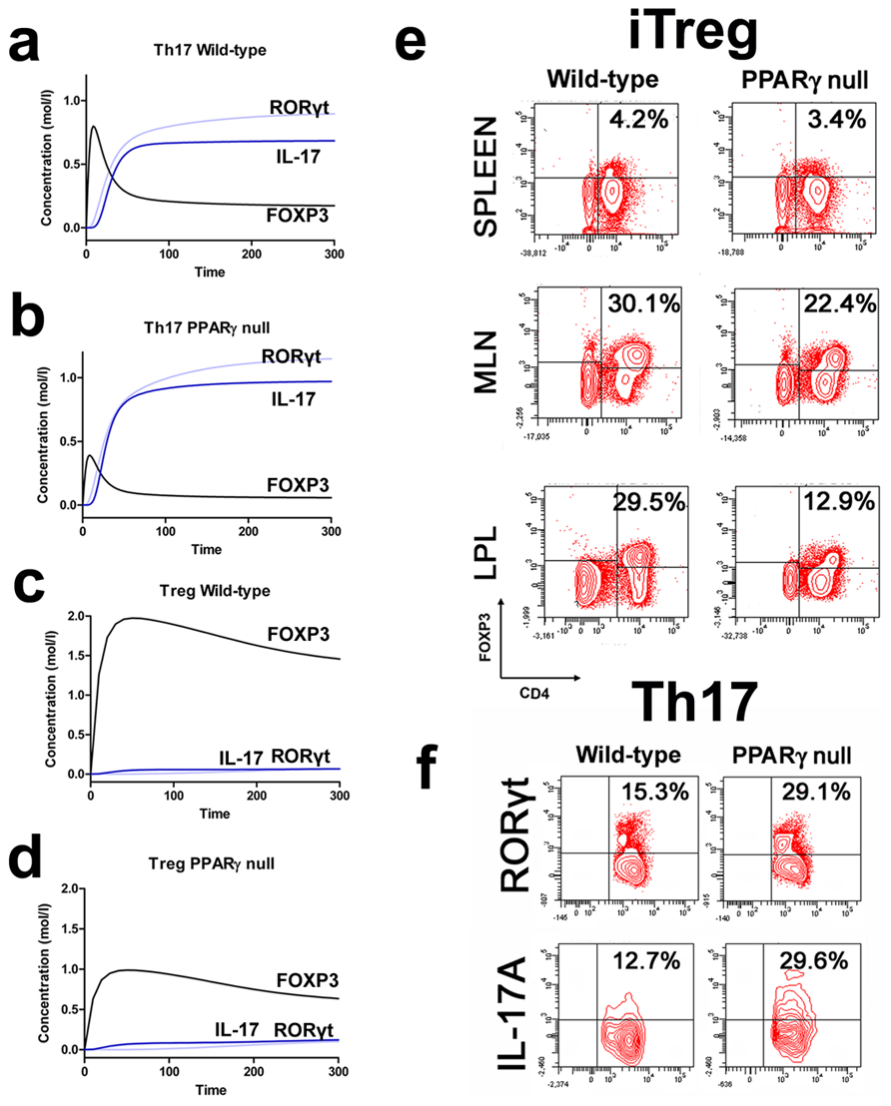
Peroxisome proliferator-activated receptor (PPAR) γ suppresses T helper (Th)17 cell differentiation and upregulates FOXP3 expression *in vivo*. (A–D) Computational simulation of the effect of PPARγ deficiency on differentiation from a naïve state into either Th17 or iTreg phenotypes. (E) Th17 cell accumulation in spleens of recipients of wild-type versus PPARγ null CD4+ T cells. (F) Treg cell accumulation in spleen, mesenteric lymph nodes (MLN) and lamina propria (LP) of SCID recipient mice.

**Figure 4:**
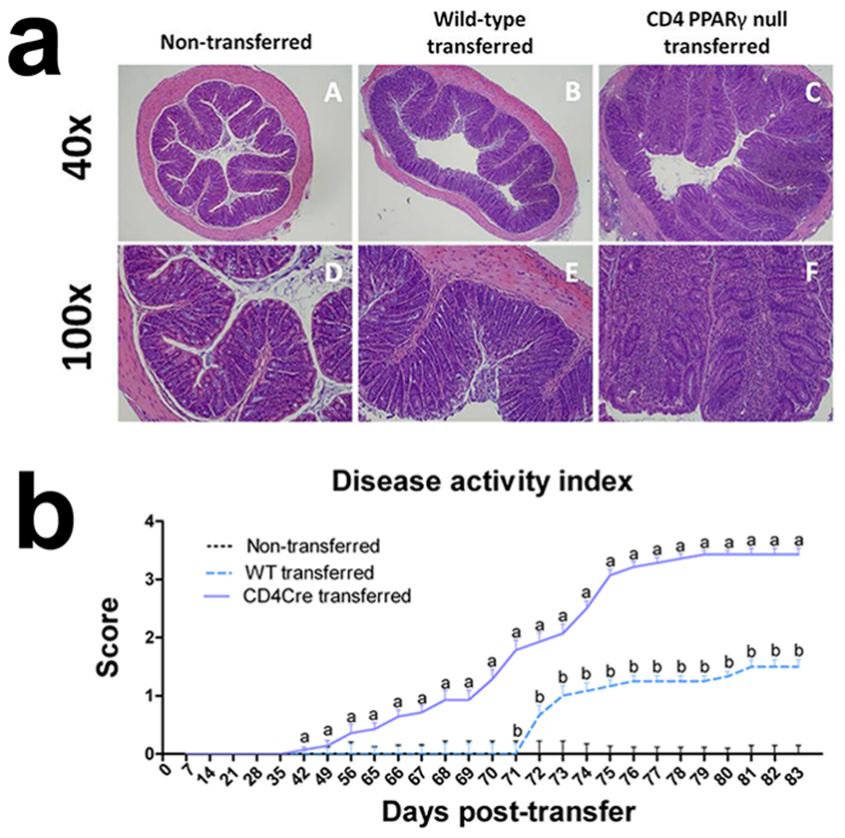
Peroxisome proliferator activated receptor gamma (PPARγ) ameliorates colonic inflammation and clinical signs of disease. (A) SCID recipients were administered either wild-type or PPARγ null naïve CD4+ T cells. Representative photomicrographs of colons from (A.A–A.D) non-transferred (A.B–A.E) wild-type recipient and (A.C,A.F) T cell-specific PPARγ null recipient mice. Original magnification 40 or 100×. (B) Disease activity scores of SCID recipient mice of wild-type or PPARγ null naïve CD4+ T cells. Data are represented as mean ± standard error. Points with an asterisk are significantly different when compared to the PBS-treated group (*P*<0.05).

To determine whether the loss of T cell PPARγ favors Th17 and impairs iTreg cell differentiation and also to assess whether T cell-extrinsic mechanisms might be affecting iTreg upregulation we conducted computational simulations and *in vivo* studies of PPARγ deletion in T cells. Chronologically, a PPARγ-deficient naïve CD4+ T cell was created *in silico* by blocking PPARγ downstream signaling. The loss of PPARγ *in silico* caused upregulation of RORγt and IL-17 in Th17 cells (Figure 3B) and down-regulation of FOXP3 in iTreg cells (Figure 3D) compared to wild-type CD4+ T cells (Figure 3A and 3C). These results demonstrate that PPARγ exerts a regulatory role in CD4+ T cell differentiation from a naïve state to Th17 or iTreg cells. Next, to validate this computational prediction, we sorted CD4+CD25-CD45RB^high^ naïve T cells from spleens of donor wild-type and T cell-specific PPARγ null mice and adoptively transferred 4×10^5^ viable cells to SCID recipients (Figure S8). Cells isolated from the colonic lamina propria (LP), spleen and mesenteric lymph nodes (MLN) of recipient mice were assayed for expression of FOXP3, RORγt and IL-17A by intracellular flow cytometry. The transfer of CD4+ T cells lacking PPARγ resulted in significantly greater accumulation of IL-17-producing Th17 cells and lower levels of FOXP3+ iTreg cells in spleen, MLN and colonic LP of recipient mice (Figure 3E and 3F and Figure S9). Recipients of PPARγ null cells showed a significantly more severe and earlier onset of disease when compared to recipients of wild-type cells (Figure 4A). Histological examination demonstrated that colons recovered from recipients of PPARγ null CD4+ T cells had significantly greater lymphocytic infiltration and crypt hyperplasia than those recovered from recipients of wild-type CD4+ T cells (Figure 4B).

### Pharmacological activation of PPARγ favors a switch of Th17 cells towards an iTreg phenotype *in vivo*

**Figure 5:**
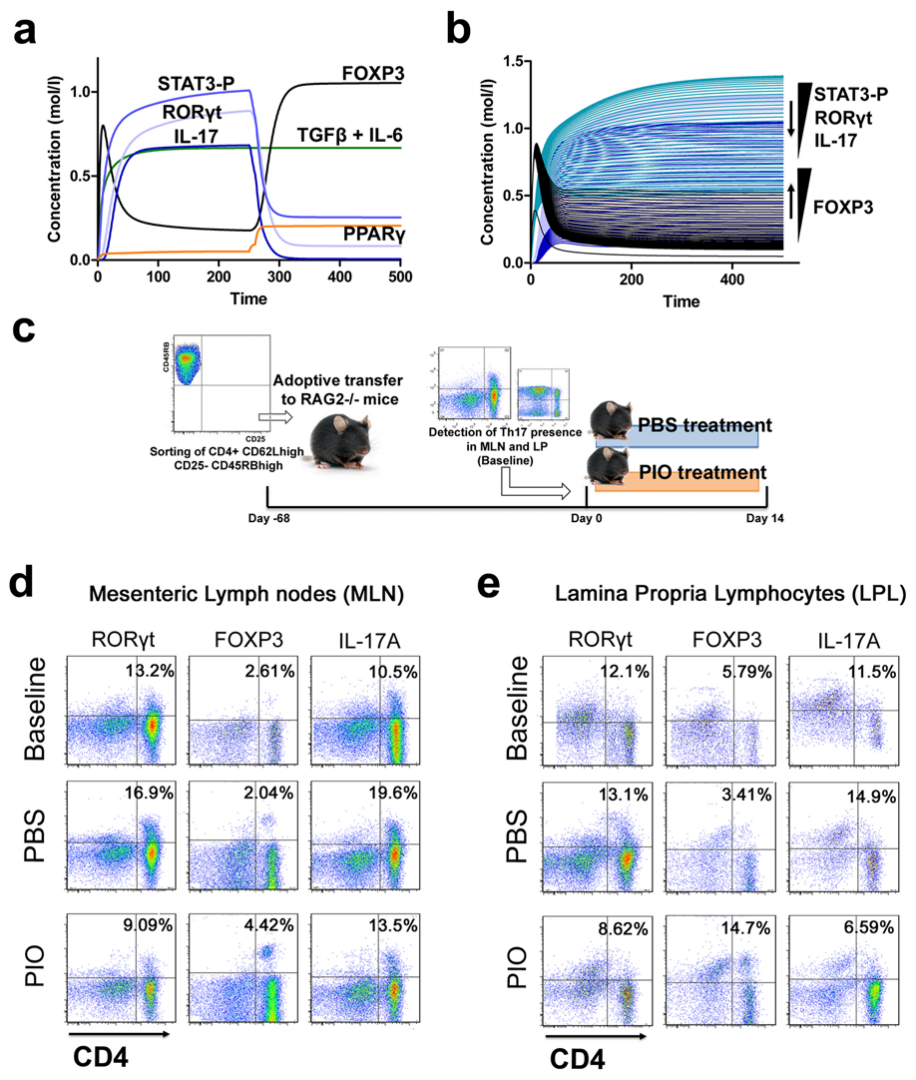
Validation of the modeling prediction regarding the role of peroxisome proliferator-activated receptor (PPAR) γ in regulating the plasticity between T helper (Th) 17 and induced regulatory T cells (iTreg). (A) Computer simulation illustrating a down-modulation of IL-17, STAT3, RORγt and upregulation of FOXP3 in a differentiated Th17 cell following PPARγ activation. (B) Combination of time-course and PPARγ concentration scan to assess changes of IL-17, STAT3, RORγt and FOXP3 over time. (C) Experimental design for the validation of the model prediction. (D–E) Accumulation of iTreg and Th17 cells in the mesenteric lymph nodes (MLN) and colonic lamina propria (LP) of recipient mice.

To determine whether PPARγ activation played an essential role in converting fully differentiated Th17 cells into iTreg cells, the computational model was induced to Th17 with the addition of IL-6 and TGFβ and PPARγ was activated when the cell was a fully differentiated Th17. Results show that following induction of Th17 and subsequent PPARγ activation, IL-17, STAT-3 and RORγt were dramatically downregulated, whereas FOXP3 was upregulated, thereby demonstrating a phenotypic switch from a Th17 to an iTreg phenotype (Figure 5A). To ensure that parameter space scan and time-course were linked and the changes in PPARγ were being observed in a time-dependent manner, a combination of both was run, reiterating the phenotype switch with increasing concentrations of PPARγ over time observing an upregulation of FOXP3 and a downregulation of IL-17, RORγt and STAT3-P (Figure 5B). To address this hypothesis, we sorted CD4+ CD25- CD45RB^high^ naïve T cells from spleens of donor wild-type mice and transferred 4×10^5^ viable cells to RAG2^−/−^ recipients. When clinical signs of disease and colitis appeared, a subset of mice was sacrificed and spleen, MLN and colons were extracted to examine Th17 and Treg levels (baseline results). After verifying the presence of Th17 cells in colon, MLN and spleen, half of the mice were received a daily treatment of 70 mg/kg of pioglitazone given orally to activate PPARγ (Figure 5C). During the treatment period, mice treated with pioglitazone recovered weight and their disease activity scores dropped significantly (Figure S10) compared to mice treated with PBS (Figure S11). Histopathological examinations also showed that colons from recipient mice treated with pioglitazone had a significantly lower lymphocytic infiltration and crypt hyperplasia than those from non-treated recipients (Figure S12). Untreated mice maintained a predominant Th17 response characterized by increased levels of CD4+ T cells expressing RORγt and IL-17A. In contrast, pioglitazone-treated mice not only recovered from colitis and its associated weight loss, but also showed a switch from a predominant Th17 into an iTreg phenotype characterized by increased expression of FOXP3 and decreased IL17-A and RORγt in CD4+ T cells of the colonic LP and MLN (Figure 5D and 5E and Figure S13). This data supports the *in silico* prediction that activation of PPARγ in Th17 cells favors differentiation into iTreg cells, which facilitates colonic tissue reconstitution and recovery from disease.

## Discussion

Computational models can help to synthesize and integrate existing knowledge and narrow the experimental design prior to costly *in vivo* experimentation. To gain a more comprehensive understanding of the mechanisms controlling CD4+ T cell differentiation, we first compiled and integrated existing literature knowledge and data related to the cytokines and intracellular signaling pathways involved in the differentiation of a naïve CD4+ T cell into effector and regulatory cell subsets. To determine whether the model predictions regarding novel mechanisms of immunoregulation in Th17 and Treg cells were sensitive to the model parameters we performed a sensitivity analysis of the signaling pathways controlling Th17 and iTreg phenotypes. Our simulations reproduced known CD4+ T cell differentiation behaviors for Th1, Th2, Th17 and iTreg, and predicted novel mechanisms of T cell-mediated immunoregulation. By simulating the cytokine milieu that surrounds a CD4+ T cell *in silico*, we dissected crucial signaling pathways and their transcriptional regulation programs involved in differentiation and plasticity of CD4+ T cells. While computational predictions carry certain uncertainty given by the topology of the network, computational modeling approaches applied to CD4+ T cell differentiation have proven useful in characterizing the importance of dual waves of expression of T-bet and sequentially acting positive feedback loops of TCR-IFNγ-STAT1-Tbet and IL-12-STAT4-Tbet signaling in Th1 differentiation [32]. A central question in T cell biology involves improving the understanding of instructive versus selective factors that regulate the differentiation process. Selective factors include competition for cytokines by competing clones of CD4+ T cells in an expanding population. For example, regulatory T cells are able to outcompete for IL-2 and deprive effector T cells of this survival signal [33]. While the computational model presented herein comprehensively addresses the instructive factors (i.e., the impact of cytokine combinations on T cell phenotypes), stochastic simulations and multiscale modeling are needed to adequately model selective factors by linking molecular-level intracellular signaling sub-models and tissue-level cell-cell interaction models. Some studies have addressed selective factors by focusing on the crosstalk in molecular pathways in an expanding Th1 population using *in vitro* data [34] but only one phenotype has been computed and with a limited scope. The study presented here is the first to comprehensively investigate at the systems level the mechanisms controlling CD4+ T cell differentiation and plasticity between Th17 and iTreg cells, presenting a model that computes not only one but four of the CD4+ T cell phenotypes.

Several distinct signals regulate CD4+ T cell activation and differentiation: a signal from the T cell receptor (TCR) interacting with MHC, a co-stimulatory signal (i.e., CD28 interacting with B7.1 or B7.2 on antigen presenting cells), and a cytokine-driven signal. Other studies have more narrowly focused on CD4+ T cell proliferation [35], TCR signaling [36] or co-stimulatory signals [37]. Our mathematical approach more comprehensively studies the non-cognate interactions (i.e., cytokine milieu) and instructive factors controlling CD4+ T cell differentiation. Future studies will leverage the modeling efforts described here to construct multi-scale hybrid models driven by high-performance computing strategies that integrate sub-models of intracellular signaling pathways such as the CD4+ T cell model and tissue-level models that can simulate cell-cell interactions. These integrative approaches will provide an avenue for incorporating stochasticity as well as the modulation of phenotype and function of immune cells at sites of inflammation or infection by selective and instructive factors.

Sensitivity analyses and computational simulations using the CD4+ T cell differentiation model predicted that the nuclear receptor PPARγ modulates the balance between Th17 and iTreg cells, by controlling both the initial differentiation from a naïve CD4+ T cell as well as plasticity between phenotypes. Activation of PPARγ *in silico* favored differentiation of iTreg and antagonized Th17 differentiation by down-modulating RORγt and IL-17. These findings are in line with previous reports demonstrating that the pharmacologic activation of PPARγ selectively controls Th17 differentiation in mice and humans by interfering with RORγt transcription [38]. Furthermore, ciglitazone, a PPARγ agonist, significantly enhanced generation of iTreg cells [26] and PPARγ induced potent and stable FOXP3 expression [27] resulting in the suppression of effector CD4+ T cell responses [28]. Our *in silico* results demonstrate that the upregulation of FOXP3 and downregulation of RORγt and IL-17 in CD4+ T cells is modulated by PPARγ and behaves in a dose-dependent manner. Indeed, our *in vitro* results support the dose-dependent effect in the suppression of Th17, although not accompanied by a similar increase in FOXP3+ iTreg cells. However, our *in vivo* findings further demonstrate that pioglitazone treatment favors a switch of fully differentiated Th17 cells into an iTreg phenotype by increasing activation of PPARγ. Thus, our plasticity modeling efforts are more predictive of *in vivo* than *in vitro* behaviors of CD4+ T cells, suggesting a missing component, possibly provided by APCs in the widely utilized *in vitro* system. For instance, all trans retinoic acid, which *in vivo* is produced by APC-derived, increased and maintained FOXP3 expression [39]. Conclusively, the mechanisms by which T cell extrinsic factors modulate CD4+ T cell plasticity are yet not fully understood. Here, however, we propose PPARγ as a novel candidate for such modulation.

The CD4+ T cell mathematical model predicted an upregulation of RORγt and IL-17 in Th17 cells lacking PPARγ when compared to the wild-type counterparts. In complete correspondence to this modeling prediction, our *in vitro* results show that following Th17 differentiation, CD4+ T cells lacking PPARγ exhibit a more dramatic upregulation of RORγt and IL-17A than wild-type cells. Also, we have also observed a marginal upregulation of FOXP3 in wild-type cells. The uncoupling between the dramatic downregulation of RORγt and the more limited upregulation of FOXP3 observed *in vitro* could be attributed to external factors that play an important role in this process, which are not fully mechanistically understood or not included in the *in vitro* system used (i.e., APCs). As opposed to the *in vitro* results, the *in vivo* findings in mice with CD4+ T cell-induced colitis were more consistent with the modeling predictions. Recent studies show that changes in the cytokine environment mediate the conversion of iTreg into Th17 cells [6]. Notably, different subsets of myeloid cells in humans can orchestrate the differentiation of naïve CD4+ T cells into either effector or regulatory phenotypes [7]. Myeloid APCs are essential for the induction of IL-17A+ FOXP3+ T cells from memory CCR6+ T cells or Treg cells [40]. At the colonic mucosa, numbers and functions of IL-17-producing cells are tightly controlled by PPARγ, and its modulation of the dual roles of Th17 cells as effectors of pathogenic, tissue-damaging versus pathogen-clearing responses has been investigated in the context of *Clostridium difficile* and enteroaggregative *Escherichia coli* infections [41], [42]. However, the mechanisms controlling CD4+ T cell plasticity at the gut mucosa remain largely unknown, including the essential and dispensable regulators of these processes.

Herein, we combined computational and experimental approaches to investigate for the first time the role of PPARγ in the re-programming of fully differentiated Th17 cells into an iTreg phenotype in the gut mucosa. Of note, the presence of FOXP3 RORγt double-positive cells with suppressive actions on effector CD4+ T cell subsets has been associated with the plasticity of Th17 and iTreg [18]. TGF-β is a common inductor of Th17 and iTreg that can upregulate FOXP3, but in combination with IL-6, it upregulates IL-17 and dramatically downregulates FOXP3 expression [2]. Other cytokines, such as IL-23, modulate plasticity by restraining FOXP3+ Treg activity [14]. Clinically, inhibition of IL-17 promotes differentiation of stable iTreg cells in patients with autoimmune hepatitis [43]. However, IL-17+FOXP3+ cells were identified in inflamed intestinal mucosa of patients with Crohn's disease (CD), but not in patients with ulcerative colitis (UC) [44], the two clinical manifestations of inflammatory bowel disease. Furthermore, in line with our sensitivity analysis and computer simulations, results of our adoptive transfer studies in mice indicate that activation of PPARγ by oral pioglitazone administration favors a switch from Th17 to iTreg in MLN and colonic LP of mice with CD4+ T cell-induced colitis, thereby demonstrating that PPARγ is implicated in the modulation of CD4+ T cell plasticity *in vivo*.

The loss of PPARγ favored Th17 differentiation and reduced the conversion of IL-17A-producing Th17 cells into CD4+FOXP3+ T cells *in vivo*. Adoptive transfer studies using T cell-specific PPARγ null naïve T cells demonstrate that PPARγ is needed for suppressing effector responses at sites of inflammation such as the colonic LP in a mouse model of chronic colitis. Interestingly, FOXP3 inhibits Th17 by antagonizing the function of the transcription factors RORγt and RORα [6], [19]. This suggests a potential interaction of RORγt with FOXP3 in larger transcriptional complexes, which could explain why RORγt is more rapidly down-regulated than FOXP3 is increased. More specifically, the decrease of RORγt could result from a synergism between the inhibition exerted by PPARγ and the parallel inhibition caused by FOXP3, which in turn is enhanced when PPARγ is activated. The observation that PPARγ may interact with FOXP3 and RORγt suggests a cross-talk between transcriptional programs of crucial importance to the regulation of immune responses and clinical outcomes during infectious and immune-mediated diseases.

In summary, we demonstrate for the first time that activation of PPARγ results in reprogramming of the CD4+ T cell molecular pathways that control the Th17 phenotype, leading to the induction of an iTreg phenotype. This phenotype switch is associated with protection from CD4+ T cell-induced colitis during adoptive transfer experiments in mice. Thus, the balance between Th17 and Treg cells helps delineate the outcome of immunological processes from effector inflammation to regulatory tolerance. Our modeling approaches allowed us to narrow the design of experiments and to better understand the molecular mechanisms of action controlling CD4+ differentiation. This new mechanistic knowledge is broadly applicable to the development of immune therapeutics for infectious, allergic and immune-mediated diseases. More specifically, we propose that PPARγ is a promising therapeutic target for chronic inflammatory and infectious diseases where Th17 cells contribute to the gut immunopathogenesis.

## Materials and Methods

### Ethics statement

All experimental protocols were approved by the Virginia Tech institutional animal care and use committee (IACUC) (Protocol Number: 10-087VBI) and met or exceeded guidelines of the National Institutes of Health Office of Laboratory Animal Welfare and Public Health Service policy. Animals were under strict monitoring throughout the duration of the project and all efforts were made to minimize unnecessary pain and distress. Mice were euthanized by carbon dioxide narcosis followed by secondary cervical dislocation.

### Mathematical modeling

To facilitate a comprehensive representation of the dynamics associated with the major non-cognate pathways controlling CD4+ T cell differentiation and plasticity, we constructed an ordinary differential equation (ODE)-based computational model of the cytokines, receptors and transcription factors controlling CD4+ T cell differentiation and plasticity (Figure 1, Text S1). The mathematical model was engineered to ensure proper modulation of intracellular pathways and cell phenotypes via external cytokines representing the cytokine milieu. The mathematical model constructed was based on experimental findings and illustrates intracellular pathways controlling a naïve T cell differentiation into Th1, Th2, Th17 or iTreg phenotypes. The model comprises 60 differential equations representing 52 reactions and 93 species (Figure S3). The COmplex PAthway SImulator software [45] (COPASI; http://www.modelingimmunity.org/) was used for model development, sensitivity analysis, and calibration. Sensitivities of the steady-state fluxes of reactions were derived with respect to the reaction rates in the system. These sensitivities were normalized and represented flux control coefficients according to Metabolic Control Analysis (MCA) [46], [47]. In this case, sensitivities were performed with respect to PPARγ pathway-controlling parameters and levels of different species were assessed. The model was calibrated to experimental data (Table S1), which varied external concentration of cytokines and resulted in different phenotypes described by varying levels of transcription factors and proteins. We used the ParticleSwarm algorithm implemented in COPASI to determine unknown model parameter values and fully calibrate the model (Table S2 and S3, Figure S4). The resulting model adequately computes the differentiation of CD4+ T cells into the four phenotypes: Th1 with external IFNγ, IL-12, IL-18 and αIL-4 addition, Th2 with IL-4 and αIFNγ addition and iTreg with IL-2 and external TGFβ addition (Figure S5). Also, to induce Th17 differentiation from a naïve CD4+ T cell, external IL-6 and external TGF-β were added in combination and demonstrated upregulation of RORγt, IL-17 and STAT-3. *In silico* simulation consisted of time-courses or parameter scans. Also, the combination of both was performed. In this last case, each plotted line has an incremented concentration of the parameter being scanned. Thus, differential patterns of expression of molecules, either upregulated or downregulation, over time can be observed by looking at the arrows in each molecule. This model is available at www.modelingimmunity.org and model assumptions and model predictions are available in the supplementary materials (Table S4 and Table S6 respectively). Also a complete table with all the numerical values of all parameters of the model is provided in the supplementary materials (Table S7).

### Mice

B6.CB17-Prkdcscid/SzJ (SCID), B6.129P2(Cg)-Rorctm2Litt/J, C57BL/6J and B6(Cg)-Rag2tm1.1Cgn/J were purchased from The Jackson Laboratory and housed under specific pathogen-free conditions in ventilated racks. The mice were maintained in the animal facilities at Virginia Tech. All experimental protocols were approved by the institutional animal care and use committee at Virginia Tech and met or exceeded guidelines of the National Institutes of Health Office of Laboratory Animal Welfare and Public Health Service policy.

### Cell isolation

Spleens and mesenteric lymph nodes (MLN) were excised and crushed in 1×PBS/5% FBS using the frosted ends of two sterile microscope slides. Single cell suspensions were centrifuged at 300× g for 10 min and washed once with 1×PBS. Red blood cells were removed by osmotic lysis prior to the washing step. All cell pellets were resuspended in FACS buffer (1×PBS supplemented with 5% FBS and 0.09% sodium azide) and subjected to flow cytometric analysis. Paralelly, colons were excised and lamina propria leukocytes (LPL) were isolated. Tissue pieces were washed in CMF (1× HBSS/10% FBS/25 mM Hepes), and tissue was incubated twice with CMF/5 mM EDTA for 15 min at 37°C while stirring. After washing with 1×PBS, tissue was further digested in CMF supplemented with 300 U/ml type VIII collagenase and 50 U/ml DNAse I (both Sigma-Aldrich) for 1.5 hs at 37°C while stirring. After filtering the supernatants, cells were washed once in 1×PBS, pellets were resuspended in FACS buffer and subjected to flow cytometric analysis.

### Immunophenotyping and cytokine analysis by flow cytometry

For fluorescent staining of immune cell subsets 4–6×10^5^ cells were incubated for 20 min with fluorochrome-conjugated primary mouse specific antibodies: anti-CD3 PE-Cy5 clone 145-2C11 (eBioscience), anti-CD4 PE-Cy7 clone GK1.5 (eBioscience), anti-CD4 APC clone RM4-5 and anti-CD25 Biotin clone 7D4 (BD Biosciences). Cells were washed with FACS buffer (1×PBS supplemented with 5% FBS and 0.09% sodium azide). For intracellular staining of transcription factors and cytokines, cells were fixed and permeabilized using a commercial kit according to the manufacturer's instructions (eBioscience). Briefly, cells were fixed and permeabilized for 20 minutes, Fc receptors were blocked with mouse anti-CD16/CD32 FcBlock (BD Biosciences) and cells were stained with fluorochrome-conjugated antibodies towards anti-mouse, FOXP3 FITC clone FJK-16s, anti-mouse ROR gamma (t) PE, clone B2B and anti-mouse IL17-A APC, clone eBio17B7 (eBioscience). All samples were stored fixed at 4°C in the dark until acquisition on a LSR II flow cytometer (BD Biosciences). A live cell gate (FSC-A, SSC-A) was applied to all samples followed by single cell gating (FSC-H, FSC-W) before cells were analyzed for the expression of specific markers. Data analysis was performed with FACS Diva (BD Biosciences) and Flow Jo (Tree Star Inc.).

### Adoptive transfer studies in mice

Six-week-old SCID and RAG2-/- mice were administered intraperitoneally (i.p.) 4×10^5^ CD4+ CD45RB^high^ CD25- from either CD4 null PPAR γ fl/fl or C57BL/6J (wild-type), or B6.129P2(Cg)-Rorctm2Litt/J mice. Mice were weighed on a weekly basis and clinical signs of disease were recorded daily for 14 wk. Mice that developed severe signs of wasting disease were sacrificed. Otherwise, mice were sacrificed 90 days after transfer.

### CD4+ T cell subset sorting

Splenocytes obtained from CD4 null PPAR-γ fl/fl or C57BL/6J (wild-type) mice were enriched in CD4+ T cells by magnetic negative sorting using the I-Mag cell separation system (BD Pharmingen). Cells were incubated with a mixture of biotinylated Abs followed by a second incubation with streptavidin particles and exposed to a magnet to remove unwanted cells. The purity of the CD4+-enriched cell suspension was between 93 and 96%. CD4-enriched cells were used for adoptive transfer, or further purified by FACS. For FACS sorting, cells were labeled with CD45RB, CD4, and CD25 and separated into CD4+ CD45RB^high^ CD25- cells (i.e., effector T cells) in a FACSAria cell sorter (BD Biosciences). The purity of the FACS-sorted CD4+ subsets was ≥98%.

### *In vitro* CD4+ T cell differentiation studies

CD4+CD62L+ cells from either wild-type or T PPARγ null (CD4Cre+) mice were sorted using magnetic activated cell sorting (MACS, Miltenyi Biotec) and stimulated with plate bound anti-CD3 (5 µg/ml, BD Biosciences) under Th17 conditions with 2.5 ng/ml hTGF-β1 (R&D Systems), 25 ng/ml IL-6 (Peprotech), 10 µg/ml anti-IL-4 (clone 11B11, R&D Systems), and 10 µg/ml anti-IFN-γ (clone XMG1.2, R&D Systems). 60 hours after activation, an aliquot was obtained to check purity and DMSO-diluted pioglitazone (PIO, Cayman Chemicals) was added to the media at 0, 0.1, 1, 10, 40 or 80 µM. Control (0 µM PIO) was treated with DMSO only. 24 hours after treatment Th17 cells were restimulated with PMA (50 ng/mL, Acros Organics) and ionomycin (500 ng/mL, Sigma) in the presence of BD GolgiStop (BD Biosciences) for 6 h, after which intracellular staining was performed. The experiment was repeated three times for consistency. Co-stimulation of with CD28 has been described to downregulate Th17 development [37], [48]. We also performed optimization studies for Th17 differentiation using CD28 as a co-stimulatory signal and the addition of recombinant IL-23 in the cytokine cocktail, however, no differences were observed. Co-stimulation signaling optimization studies were run adding either 0 or 2.5 µg/mL of αCD28 in the media. No differences were found. Thus, the data presented are with αCD3 stimulation only.

### Histopathology

Colonic sections were fixed in 10% buffered neutral formalin, later embedded in paraffin and then sectioned (5 µm) and stained with H&E stain for histological examination. Colons were graded with a compounded histological score including the extent of (1) leukocyte infiltration, (2) mucosal thickening and (3) epithelial cell erosion. The sections were graded with a score of 0–4 for each of the previous categories, and data were analyzed as a normalized compounded score.

### Statistical analysis

Parametric data were analyzed using the ANOVA followed by Scheffe's multiple comparison method. Nonparametric data were analyzed by using the Mann-Whitney's *U* test followed by a Dunn's multiple comparisons test. ANOVA was performed by using the general linear model procedure of SAS, release 6.0.3 (SAS Institute). Statistical significance was assessed at a *P*≤0.05.

## Supporting Information

Figure S1 **Schematic representation of the cytokines and transcription factors controlling CD4+ T cell differentiation.** Our CD4+ T cell differentiation model is firmly grounded on experimental observations and reproduces four CD4+ T cell phenotypes upon external stimulation with appropriate cytokine combinations, as well as representing the crosstalk between phenotypes, exhibiting inhibitory trends.
(TIF)

Figure S2 **Iterative systems modeling approaches used by the Center of Modeling Immunity to Enteric Pathogens (MIEP) program (** www.modelingimmunity.org **).** The modeling approaches include fully integrated computational strategies and experimental validation studies. After literature search and generation of calibration data, a comprehensive network is created using CellDesigner. Parameters are then adjusted in the model using the modeling software COmplex PAthway SImulator (COPASI) and quality control analysis is performed. *In silico* experimentation is conducted and several hypotheses are generated. These hypotheses will then be tested using in *vivo* and *in vitro* experimentation. Finally, the new data generated will be used to re-calibrate the model to start the process again.
(TIF)

Figure S3 **Ordinary Differential Equations (ODE) triggering activation and inhibition regulatory and effector pathways in our CD4+ T cell model.** Briefly, mass action and the Hill functions were used to reproduce CD4+ T cell behaviors *in silico* based on initial stimulation by external cytokines.
(PDF)

Figure S4 **Parameter estimation results for the Th17 phenotype.** IL-17 and FOXP3 were fitted by COPASI using the ParticleSwarm algorithm. The fitted value (dark blue and pink dots) could reproduce the behavior of the measured value (red and light blue dots). The weighted error (green dots) is around 0, indicating that the fitting has been performed successfully.
(TIF)

Figure S5 **Induction of effector T helper type 1 (Th1), type 2 (Th2), type 17 (Th17) and induced regulatory T cell (iTreg) phenotype differentiation** ***in silico*** **.** The addition of increasing amounts of IL-12, IL-18 and IFN-γ (Th1), IL-4 (Th2), IL-6 and TGF-β (Th17) or TGF-β alone (iTreg) as external stimuli in the system resulted in increasing amounts of related molecules for each phenotype.
(TIF)

Figure S6 **Sensitivity analysis on peroxisome proliferator-activated receptor γ (PPARγ) by the CD4+ T cell computational model.** Sensitivity analysis was run with COPASI on our computational model using a delta factor of 0.0001 and a delta minimum of 1e-12. The subtask run for the analysis was a time-series with t = 100 h and correlation of all the variables of the model against activated PPARγ was assessed, showing high correlation with key transcription factors that determine phenotype differentiation on Th17 and iTreg.
(TIF)

Figure S7 **Effect of peroxisome proliferator-activated receptor γ (PPARγ) on T helper (Th)17 and induced regulatory T cell (iTreg) markers** ***in vitro*** **.** (**A**) Increasing concentrations of pioglitazone (PIO), a full PPARγ agonist, upregulate FOXP3 in wild-type Th17 differentiated cells following 24 h treatment and down-regulate RORγt and IL-17A in wild-type cells. (**B**) Increasing concentrations of PIO do not have an effect in PPARγ null Th17 cells. Data are represented as mean ± standard error. Points with an asterisk are significantly different when comparing different PIO treatments with to the non-treated group (*P*<0.05).
(TIF)

Figure S8 **Experimental design to validate peroxisome proliferator-activated receptor γ (PPARγ) knockout predictions by the CD4+ T cell computational model.** Wild-type or PPARγ null splenocytes were isolated and CD4+ enriched to then sort naïve CD4+ T cells and transfer them into a SCID mouse to assess PPARγ-related patterns of differentiation.
(TIF)

Figure S9 **Effect of peroxisome proliferator-activated receptor γ (PPARγ) on T helper (Th)17 and induced regulatory T cell (iTreg) markers** ***in vivo*** **.** (A) Treg cell accumulation in spleen, mesenteric lymph nodes (MLN) and lamina propria (LP) of SCID recipient mice. (B) Th17 cell accumulation in spleens of recipients of wild-type versus PPARγ null CD4+ T cells. Data are represented as mean ± standard error. Points with an asterisk are significantly different when comparing the PPARγ null group to the wild-type group (*P*<0.05).
(TIF)

Figure S10 **Improvement in Disease Activity Index (DAI) following oral treatment with pioglitazone (PIO) in RAG2-/- mice.** RAG2-/- adoptive transfer recipient mice were treated with either PIO or PBS (control group) and given a composite score reflecting clinical signs of the disease (i.e. perianal soiling, rectal bleeding, diarrhea, and piloerection) for 14 days daily. Data are represented as mean ± standard error. Points with an asterisk are significantly different when comparing the PIO-treated group to the PBS-treated group (P<0.05).
(TIF)

Figure S11 **Improvement in mouse body weight following oral treatment with pioglitazone in RAG2-/- mice.** RAG2-/- adoptive transfer recipient mice were treated with either PIO or PBS (control group) for 14 days and the average daily loss in body weights throughout the 14 day treatment was calculated. Data are represented as mean ± standard error. Points with an asterisk are significantly different when compared to the PBS-treated group (P<0.05).
(TIF)

Figure S12 **Histopathological analysis on colonic tissue from adoptive transfer studies.** RAG2-/- adoptive transfer recipient mice were treated with either PIO or PBS (control group) for 14 days and histopathological assessment was performed. All specimens underwent blinded histological examination and were scored (0–4) on leukocyte infiltration (LI), epithelial erosion (EE) and mucosal wall thickening (MT) on day 14 after treatment. Data are represented as mean ± standard error. Points with an asterisk are significantly different at a given time point (P<0.05).
(TIF)

Figure S13 **Pharmacological activation of peroxisome proliferator-activated receptor γ (PPARγ) favors a switch of Th17 cells towards an iTreg phenotype** ***in vivo*** **.** RAG2-/- mice with induced chronic colitis were treated with either PBS or PIO for 14 days and flow cytometry were assessed at day 0 (baseline) and at the end of the treatment. (A) Accumulation of iTreg and Th17 cells in the mesenteric lymph nodes (MLN) (B) Accumulation of iTreg and Th17 cells in the colonic lamina propria (LP) of recipient mice. Data are represented as mean ± standard error. Points with an asterisk are significantly different at a given time point (*P*<0.05).
(TIF)

Table S1 **Calibration database to adjust parameters on the CD4+ T cell computational model.** Data is represented as either external inputs for the model or internal readings. The external cytokines (external input) will trigger different phenotype induction depending on the concentration. Consequently, different cytokines and transcription factors will be upregulated (internal readings).
(XLSX)

Table S2 **Complete assessment of 7 parameter estimations performed by using COPASI's Particle Swarm algorithm with 3000 iterations and a particle size of 50 for reactions number 10, 11, 13 and 14.** This table was used to compare turnover values as well as optimal gradients to choose an effective combination of parameters.
(XLSX)

Table S3 **CD4+ T cell model fitting performed by using COPASI's global parameter estimation.** A species is fitted computationally using experimental data and simulation algorithms. The objective value is the value that COPASI targets based on the experimental data and the computational simulation.
(XLSX)

Table S4 **Table of assumptions for the representation of activation and inhibition pathways of the CD4+ T cell computational model.** Modeling assumptions were made based on the literature and on experimental observations to be able to properly modulate and calibrate the CD4+ T cell computational model.
(XLSX)

Table S5 **Comprehensive summary of stimuli input versus molecule expression output.** The four CD4+ T cell phenotypes by a variety of external stimuli represented in the second column. These external stimuli cause upregulation of molecules represented in the third column and downregulation of the molecules represented in the fourth column.
(XLSX)

Table S6 **Table of predictions derived from the CD4+ T cell computational model.** The CD4+ T cell differentiation model predicted behaviors 1–4 at a post-calibration stage. These predictions are the results of *in silico* experimentation using scans, time-courses and loss-of-function approaches.
(XLSX)

Table S7 **Complete dynamics of the CD4+ T cell differentiation model.** Numerical values for all parameters of the model were assessed performing the computation of the ParticleSwarm algorithm in COPASI and using experimental data from the literature.
(XLSX)

Text S1 **Basic information on model creation, model calibration and simulation process.** Briefly, the model was constructed using Th1, Th2, Th17 and iTreg information from the literature. Parameter estimation was ran using the Complex Pathway Simulator (COPASI) and quality control was performed to ensure proper initialization and fate. Afterwards, in silico experimentation was run to produce computational hypotheses.
(DOCX)

## References

[1] McGeachy MJ, Bak-Jensen KS, Chen Y, Tato CM, Blumenschein W, et al (2007) TGF-beta and IL-6 drive the production of IL-17 and IL-10 by T cells and restrain T(H)-17 cell-mediated pathology. Nat Immunol 8: 1390–1397. 17994024 10.1038/ni1539

[2] Bettelli E, Carrier Y, Gao W, Korn T, Strom TB, et al (2006) Reciprocal developmental pathways for the generation of pathogenic effector TH17 and regulatory T cells. Nature 441: 235–238. 16648838 10.1038/nature04753

[3] Li H, Rostami A (2010) IL-9: basic biology, signaling pathways in CD4+ T cells and implications for autoimmunity. J Neuroimmune Pharmacol 5: 198–209. 20020328 10.1007/s11481-009-9186-y

[4] Trifari S, Spits H (2010) IL-22-producing CD4+ T cells: middle-men between the immune system and its environment. Eur J Immunol 40: 2369–2371. 20809491 10.1002/eji.201040848

[5] Levings MK, Roncarolo MG (2000) T-regulatory 1 cells: a novel subset of CD4 T cells with immunoregulatory properties. J Allergy Clin Immunol 106: S109–112. 10887343 10.1067/mai.2000.106635

[6] Yang XO, Nurieva R, Martinez GJ, Kang HS, Chung Y, et al (2008) Molecular antagonism and plasticity of regulatory and inflammatory T cell programs. Immunity 29: 44–56. 18585065 10.1016/j.immuni.2008.05.007 PMC2630532

[7] Hoechst B, Gamrekelashvili J, Manns MP, Greten TF, Korangy F (2011) Plasticity of human Th17 cells and iTregs is orchestrated by different subsets of myeloid cells. Blood 117: 6532–6541. 21493801 10.1182/blood-2010-11-317321

[8] Zhou L, Chong MM, Littman DR (2009) Plasticity of CD4+ T cell lineage differentiation. Immunity 30: 646–655. 19464987 10.1016/j.immuni.2009.05.001

[9] Hegazy AN, Peine M, Helmstetter C, Panse I, Frohlich A, et al (2010) Interferons direct Th2 cell reprogramming to generate a stable GATA-3(+)T-bet(+) cell subset with combined Th2 and Th1 cell functions. Immunity 32: 116–128. 20079668 10.1016/j.immuni.2009.12.004

[10] Cao W, Chen Y, Alkan S, Subramaniam A, Long F, et al (2005) Human T helper (Th) cell lineage commitment is not directly linked to the secretion of IFN-gamma or IL-4: characterization of Th cells isolated by FACS based on IFN-gamma and IL-4 secretion. Eur J Immunol 35: 2709–2717. 16106470 10.1002/eji.200425957

[11] Huang Z, Xin J, Coleman J, Huang H (2005) IFN-gamma suppresses STAT6 phosphorylation by inhibiting its recruitment to the IL-4 receptor. J Immunol 174: 1332–1337. 15661890 10.4049/jimmunol.174.3.1332

[12] Mendoza L (2006) A network model for the control of the differentiation process in Th cells. Biosystems 84: 101–114. 16386358 10.1016/j.biosystems.2005.10.004

[13] Nistala K, Adams S, Cambrook H, Ursu S, Olivito B, et al (2010) Th17 plasticity in human autoimmune arthritis is driven by the inflammatory environment. Proc Natl Acad Sci U S A 107: 14751–14756. 20679229 10.1073/pnas.1003852107 PMC2930428

[14] Morrison PJ, Ballantyne SJ, Kullberg MC (2011) Interleukin-23 and T helper 17-type responses in intestinal inflammation: from cytokines to T-cell plasticity. Immunology 133: 397–408. 21631495 10.1111/j.1365-2567.2011.03454.x PMC3143351

[15] Lee YK, Turner H, Maynard CL, Oliver JR, Chen D, et al (2009) Late developmental plasticity in the T helper 17 lineage. Immunity 30: 92–107. 19119024 10.1016/j.immuni.2008.11.005 PMC3607320

[16] Lopez P, Gonzalez-Rodriguez I, Gueimonde M, Margolles A, Suarez A (2011) Immune response to Bifidobacterium bifidum strains support Treg/Th17 plasticity. PLoS One 6: e24776. 21966367 10.1371/journal.pone.0024776 PMC3178565

[17] Ye J, Su X, Hsueh EC, Zhang Y, Koenig JM, et al (2011) Human tumor-infiltrating Th17 cells have the capacity to differentiate into IFN-gamma+ and FOXP3+ T cells with potent suppressive function. Eur J Immunol 41: 936–951. 21381020 10.1002/eji.201040682

[18] Tartar DM, VanMorlan AM, Wan X, Guloglu FB, Jain R, et al (2010) FoxP3+RORgammat+ T helper intermediates display suppressive function against autoimmune diabetes. J Immunol 184: 3377–3385. 20181889 10.4049/jimmunol.0903324 PMC2843758

[19] Zhou L, Lopes JE, Chong MM, Ivanov II, Min R, et al (2008) TGF-beta-induced Foxp3 inhibits T(H)17 cell differentiation by antagonizing RORgammat function. Nature 453: 236–240. 18368049 10.1038/nature06878 PMC2597437

[20] Koenen HJ, Smeets RL, Vink PM, van Rijssen E, Boots AM, et al (2008) Human CD25highFoxp3pos regulatory T cells differentiate into IL-17-producing cells. Blood 112: 2340–2352. 18617638 10.1182/blood-2008-01-133967

[21] Ivanov II, McKenzie BS, Zhou L, Tadokoro CE, Lepelley A, et al (2006) The orphan nuclear receptor RORgammat directs the differentiation program of proinflammatory IL-17+ T helper cells. Cell 126: 1121–1133. 16990136 10.1016/j.cell.2006.07.035

[22] Manel N, Unutmaz D, Littman DR (2008) The differentiation of human T(H)-17 cells requires transforming growth factor-beta and induction of the nuclear receptor RORgammat. Nat Immunol 9: 641–649. 18454151 10.1038/ni.1610 PMC2597394

[23] Solt LA, Kumar N, Nuhant P, Wang Y, Lauer JL, et al (2011) Suppression of TH17 differentiation and autoimmunity by a synthetic ROR ligand. Nature 472: 491–494. 21499262 10.1038/nature10075 PMC3148894

[24] Huh JR, Leung MW, Huang P, Ryan DA, Krout MR, et al (2011) Digoxin and its derivatives suppress TH17 cell differentiation by antagonizing RORgammat activity. Nature 472: 486–490. 21441909 10.1038/nature09978 PMC3172133

[25] Clark RB, Bishop-Bailey D, Estrada-Hernandez T, Hla T, Puddington L, et al (2000) The nuclear receptor PPAR gamma and immunoregulation: PPAR gamma mediates inhibition of helper T cell responses. J Immunol 164: 1364–1371. 10640751 10.4049/jimmunol.164.3.1364

[26] Wohlfert EA, Nichols FC, Nevius E, Clark RB (2007) Peroxisome proliferator-activated receptor gamma (PPARgamma) and immunoregulation: enhancement of regulatory T cells through PPARgamma-dependent and -independent mechanisms. J Immunol 178: 4129–4135. 17371968 10.4049/jimmunol.178.7.4129

[27] Lei J, Hasegawa H, Matsumoto T, Yasukawa M (2010) Peroxisome proliferator-activated receptor alpha and gamma agonists together with TGF-beta convert human CD4+CD25- T cells into functional Foxp3+ regulatory T cells. J Immunol 185: 7186–7198. 21057085 10.4049/jimmunol.1001437

[28] Hontecillas R, Bassaganya-Riera J (2007) Peroxisome proliferator-activated receptor gamma is required for regulatory CD4+ T cell-mediated protection against colitis. J Immunol 178: 2940–2949. 17312139 10.4049/jimmunol.178.5.2940

[29] Klotz L, Burgdorf S, Dani I, Saijo K, Flossdorf J, et al (2009) The nuclear receptor PPAR gamma selectively inhibits Th17 differentiation in a T cell-intrinsic fashion and suppresses CNS autoimmunity. J Exp Med 206: 2079–2089. 19737866 10.1084/jem.20082771 PMC2757877

[30] Karr JR, Sanghvi JC, Macklin DN, Gutschow MV, Jacobs JM, et al (2012) A whole-cell computational model predicts phenotype from genotype. Cell 150: 389–401. 22817898 10.1016/j.cell.2012.05.044 PMC3413483

[31] Goutelle S, Maurin M, Rougier F, Barbaut X, Bourguignon L, et al (2008) The Hill equation: a review of its capabilities in pharmacological modelling. Fundam Clin Pharmacol 22: 633–648. 19049668 10.1111/j.1472-8206.2008.00633.x

[32] Schulz EG, Mariani L, Radbruch A, Hofer T (2009) Sequential polarization and imprinting of type 1 T helper lymphocytes by interferon-gamma and interleukin-12. Immunity 30: 673–683. 19409816 10.1016/j.immuni.2009.03.013

[33] Feinerman O, Jentsch G, Tkach KE, Coward JW, Hathorn MM, et al (2010) Single-cell quantification of IL-2 response by effector and regulatory T cells reveals critical plasticity in immune response. Mol Syst Biol 6: 437. 21119631 10.1038/msb.2010.90 PMC3010113

[34] Klinke DJ 2nd, Cheng N, Chambers E (2012) Quantifying crosstalk among interferon-gamma, interleukin-12, and tumor necrosis factor signaling pathways within a TH1 cell model. Sci Signal 5: ra32. 22510470 10.1126/scisignal.2002657 PMC3373264

[35] Graw F, Weber KS, Allen PM, Perelson AS (2012) Dynamics of CD4+ T Cell Responses against Listeria monocytogenes. J Immunol 189: 5250–6. 23100516 10.4049/jimmunol.1200666 PMC3504191

[36] Hong T, Xing J, Li L, Tyson JJ (2011) A mathematical model for the reciprocal differentiation of T helper 17 cells and induced regulatory T cells. PLoS Comput Biol 7: e1002122. 21829337 10.1371/journal.pcbi.1002122 PMC3145653

[37] Ying H, Yang L, Qiao G, Li Z, Zhang L, et al (2010) Cutting edge: CTLA-4–B7 interaction suppresses Th17 cell differentiation. J Immunol 185: 1375–1378. 20601598 10.4049/jimmunol.0903369 PMC2915549

[38] Klotz L, Burgdorf S, Dani I, Saijo K, Flossdorf J, et al (2009) The nuclear receptor PPAR gamma selectively inhibits Th17 differentiation in a T cell-intrinsic fashion and suppresses CNS autoimmunity. J Exp Med 206: 2079–2089. 19737866 10.1084/jem.20082771 PMC2757877

[39] Lu L, Ma J, Li Z, Lan Q, Chen M, et al (2011) All-trans retinoic acid promotes TGF-beta-induced Tregs via histone modification but not DNA demethylation on Foxp3 gene locus. PLoS One 6: e24590. 21931768 10.1371/journal.pone.0024590 PMC3172235

[40] Kryczek I, Wu K, Zhao E, Wei S, Vatan L, et al (2011) IL-17+ regulatory T cells in the microenvironments of chronic inflammation and cancer. J Immunol 186: 4388–4395. 21357259 10.4049/jimmunol.1003251

[41] Viladomiu M, Hontecillas R, Pedragosa M, Carbo A, Hoops S, et al (2012) Modeling the Role of Peroxisome Proliferator-Activated Receptor gamma and MicroRNA-146 in Mucosal Immune Responses to Clostridium difficile. PLoS One 7: e47525. 23071818 10.1371/journal.pone.0047525 PMC3469550

[42] Philipson CW, Bassaganya-Riera J, Viladomiu M, Pedragosa M, Guerrant R, et al (2013) The role of peroxisome proliferator-activated receptor gamma in immune responses to enteroaggregatice Escherichia coli infection. PLoS One 8: e57812. 23469071 10.1371/journal.pone.0057812 PMC3585146

[43] Longhi MS, Liberal R, Holder B, Robson SC, Ma Y, et al (2012) Inhibition of Interleukin-17 Promotes Differentiation of CD25(-) Cells Into Stable T Regulatory Cells in Patients With Autoimmune Hepatitis. Gastroenterology 142: 1526–35.e6. 22387392 10.1053/j.gastro.2012.02.041

[44] Hovhannisyan Z, Treatman J, Littman DR, Mayer L (2011) Characterization of interleukin-17-producing regulatory T cells in inflamed intestinal mucosa from patients with inflammatory bowel diseases. Gastroenterology 140: 957–965. 21147109 10.1053/j.gastro.2010.12.002 PMC3049831

[45] Hoops S, Sahle S, Gauges R, Lee C, Pahle J, et al (2006) COPASI- A COmplex PAthway SImulator. Bioinformatics 22: 3067–3074. 17032683 10.1093/bioinformatics/btl485

[46] Heinrich R, Rapoport TA (1974) A linear steady-state treatment of enzymatic chains. General properties, control and effector strength. Eur J Biochem 42: 89–95. 4830198 10.1111/j.1432-1033.1974.tb03318.x

[47] Kacser H, Burns JA (1973) The control of flux. Symp Soc Exp Biol 27: 65–104. 4148886

[48] Bouguermouh S, Fortin G, Baba N, Rubio M, Sarfati M (2009) CD28 co-stimulation down regulates Th17 development. PLoS One 4: e5087. 19333372 10.1371/journal.pone.0005087 PMC2658739

