# Supplementary figures and images for: Systems Modeling of Molecular Mechanisms Controlling Cytokine-driven CD4+ T Cell Differentiation and Phenotype Plasticity
Source: PLoS Comput Biol. 2013 Apr 4;9(4):e1003027. doi: 10.1371/journal.pcbi.1003027 (PMC3617204; doi:10.1371/journal.pcbi.1003027)

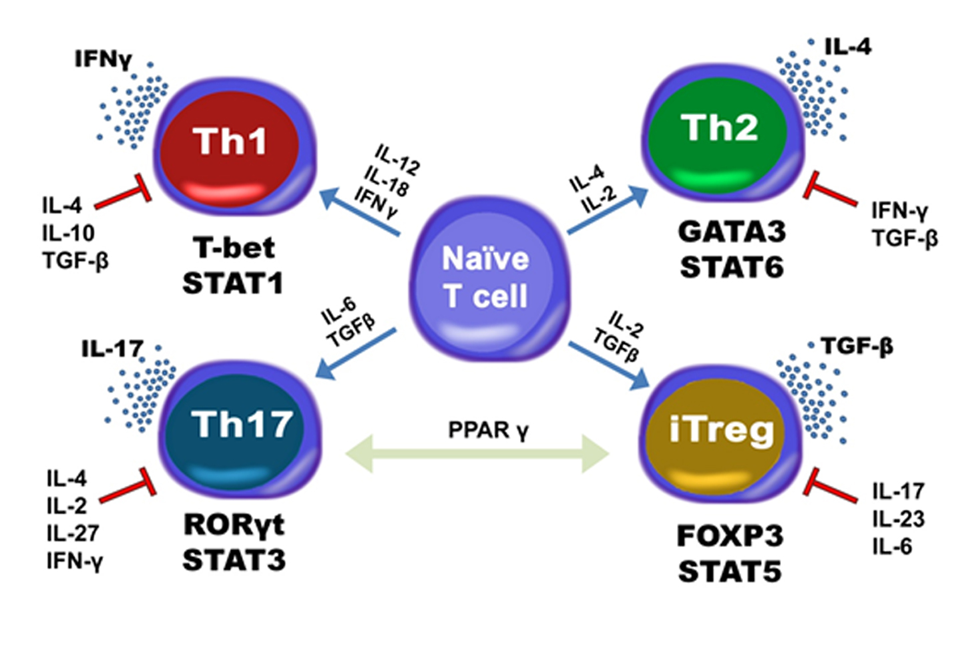

Supplement: Figure S1 — Schematic representation of the cytokines and transcription factors controlling CD4+ T cell differentiation. Our CD4+ T cell differentiation model is firmly grounded on experimental observations and reproduces four CD4+ T cell phenotypes upon external stimulation with appropriate cytokine combinations, as well as representing the crosstalk between phenotypes, exhibiting inhibitory trends. (TIF) [file pcbi.1003027.s001.tif]

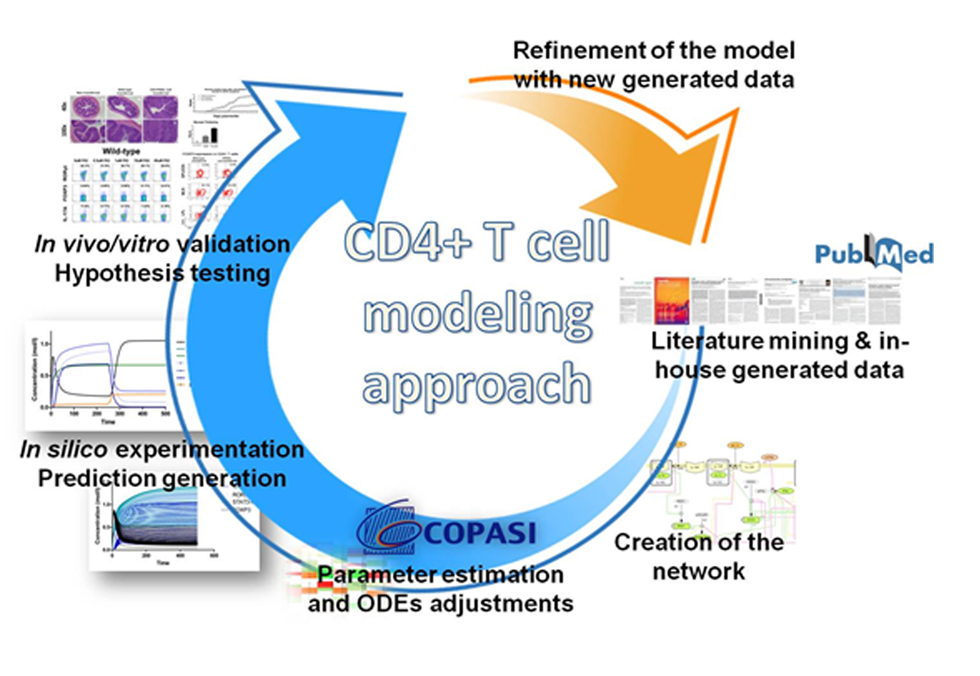

Supplement: Figure S2 — Iterative systems modeling approaches used by the Center of Modeling Immunity to Enteric Pathogens (MIEP) program ( www.modelingimmunity.org ). The modeling approaches include fully integrated computational strategies and experimental validation studies. After literature search and generation of calibration data, a comprehensive network is created using CellDesigner. Parameters are then adjusted in the model using the modeling software COmplex PAthway SImulator (COPASI) and quality control analysis is performed. In silico experimentation is conducted and several hypotheses are generated. These hypotheses will then be tested using in vivo and in vitro experimentation. Finally, the new data generated will be used to re-calibrate the model to start the process again. (TIF) [file pcbi.1003027.s002.tif]

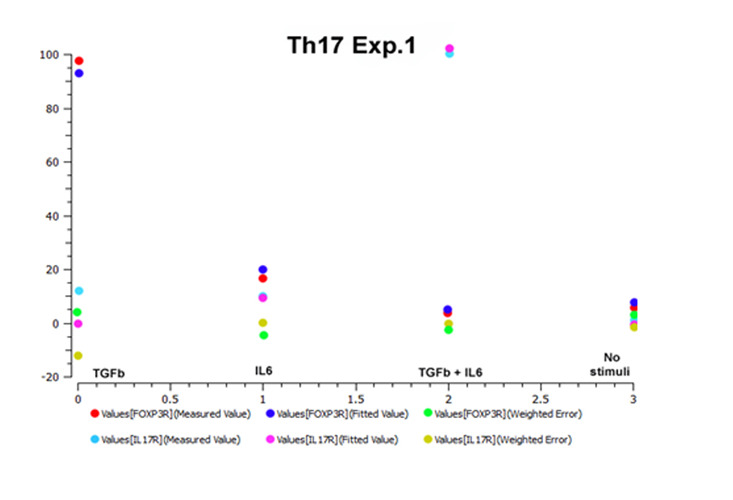

Supplement: Figure S4 — Parameter estimation results for the Th17 phenotype. IL-17 and FOXP3 were fitted by COPASI using the ParticleSwarm algorithm. The fitted value (dark blue and pink dots) could reproduce the behavior of the measured value (red and light blue dots). The weighted error (green dots) is around 0, indicating that the fitting has been performed successfully. (TIF) [file pcbi.1003027.s004.tif]

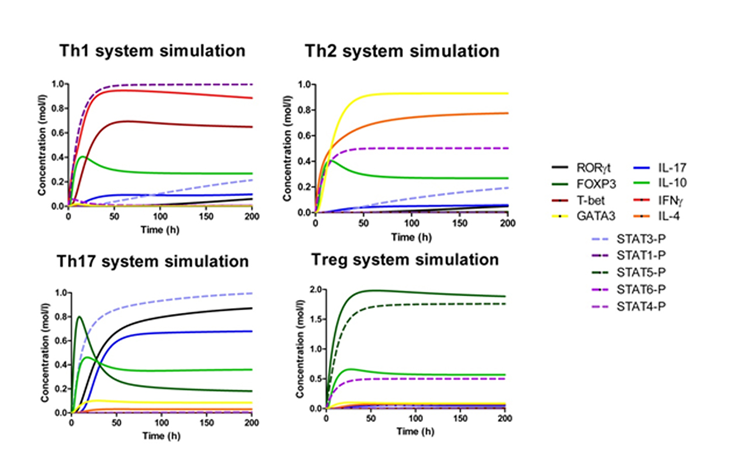

Supplement: Figure S5 — Induction of effector T helper type 1 (Th1), type 2 (Th2), type 17 (Th17) and induced regulatory T cell (iTreg) phenotype differentiation in silico . The addition of increasing amounts of IL-12, IL-18 and IFN-γ (Th1), IL-4 (Th2), IL-6 and TGF-β (Th17) or TGF-β alone (iTreg) as external stimuli in the system resulted in increasing amounts of related molecules for each phenotype. (TIF) [file pcbi.1003027.s005.tif]

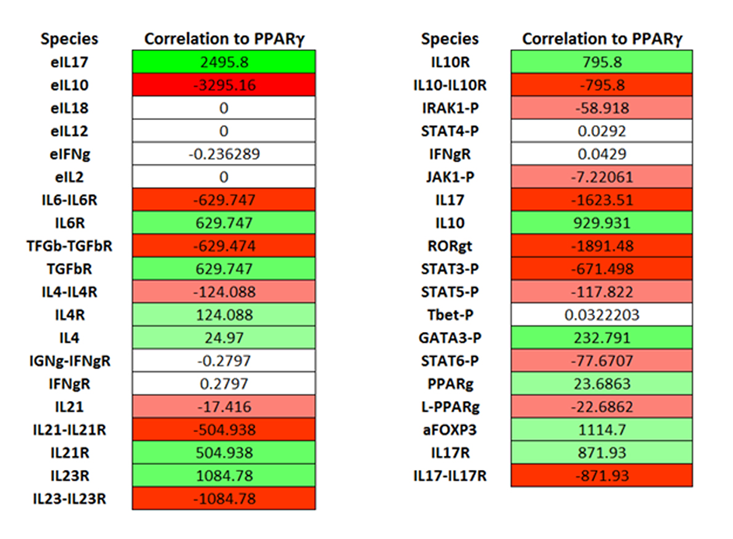

Supplement: Figure S6 — Sensitivity analysis on peroxisome proliferator-activated receptor γ (PPARγ) by the CD4+ T cell computational model. Sensitivity analysis was run with COPASI on our computational model using a delta factor of 0.0001 and a delta minimum of 1e-12. The subtask run for the analysis was a time-series with t = 100 h and correlation of all the variables of the model against activated PPARγ was assessed, showing high correlation with key transcription factors that determine phenotype differentiation on Th17 and iTreg. (TIF) [file pcbi.1003027.s006.tif]

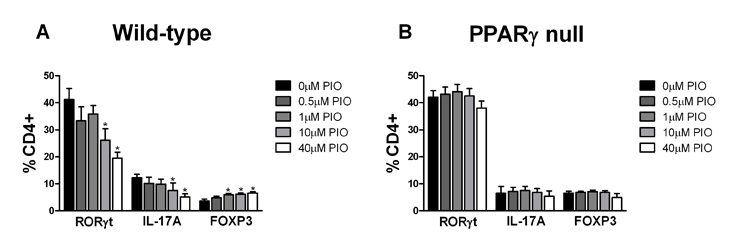

Supplement: Figure S7 — Effect of peroxisome proliferator-activated receptor γ (PPARγ) on T helper (Th)17 and induced regulatory T cell (iTreg) markers in vitro . (A) Increasing concentrations of pioglitazone (PIO), a full PPARγ agonist, upregulate FOXP3 in wild-type Th17 differentiated cells following 24 h treatment and down-regulate RORγt and IL-17A in wild-type cells. (B) Increasing concentrations of PIO do not have an effect in PPARγ null Th17 cells. Data are represented as mean ± standard error. Points with an asterisk are significantly different when comparing different PIO treatments with to the non-treated group (P<0.05). (TIF) [file pcbi.1003027.s007.tif]

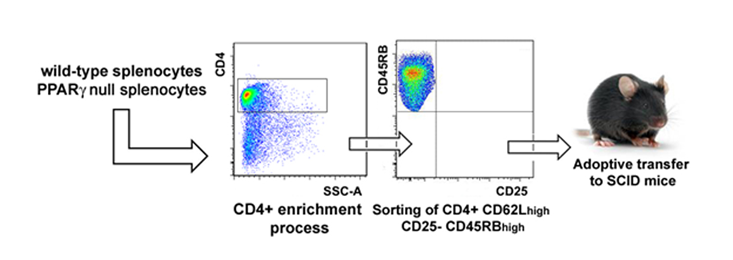

Supplement: Figure S8 — Experimental design to validate peroxisome proliferator-activated receptor γ (PPARγ) knockout predictions by the CD4+ T cell computational model. Wild-type or PPARγ null splenocytes were isolated and CD4+ enriched to then sort naïve CD4+ T cells and transfer them into a SCID mouse to assess PPARγ-related patterns of differentiation. (TIF) [file pcbi.1003027.s008.tif]

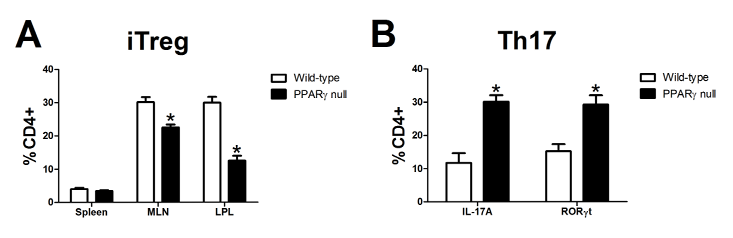

Supplement: Figure S9 — Effect of peroxisome proliferator-activated receptor γ (PPARγ) on T helper (Th)17 and induced regulatory T cell (iTreg) markers in vivo . (A) Treg cell accumulation in spleen, mesenteric lymph nodes (MLN) and lamina propria (LP) of SCID recipient mice. (B) Th17 cell accumulation in spleens of recipients of wild-type versus PPARγ null CD4+ T cells. Data are represented as mean ± standard error. Points with an asterisk are significantly different when comparing the PPARγ null group to the wild-type group (P<0.05). (TIF) [file pcbi.1003027.s009.tif]

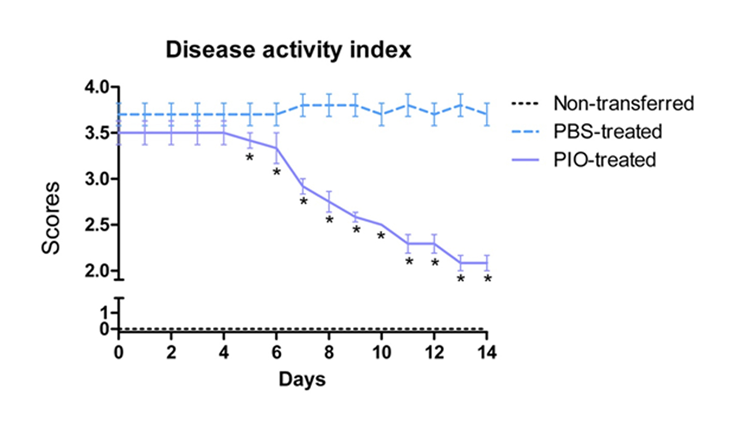

Supplement: Figure S10 — Improvement in Disease Activity Index (DAI) following oral treatment with pioglitazone (PIO) in RAG2-/- mice. RAG2-/- adoptive transfer recipient mice were treated with either PIO or PBS (control group) and given a composite score reflecting clinical signs of the disease (i.e. perianal soiling, rectal bleeding, diarrhea, and piloerection) for 14 days daily. Data are represented as mean ± standard error. Points with an asterisk are significantly different when comparing the PIO-treated group to the PBS-treated group (P<0.05). (TIF) [file pcbi.1003027.s010.tif]

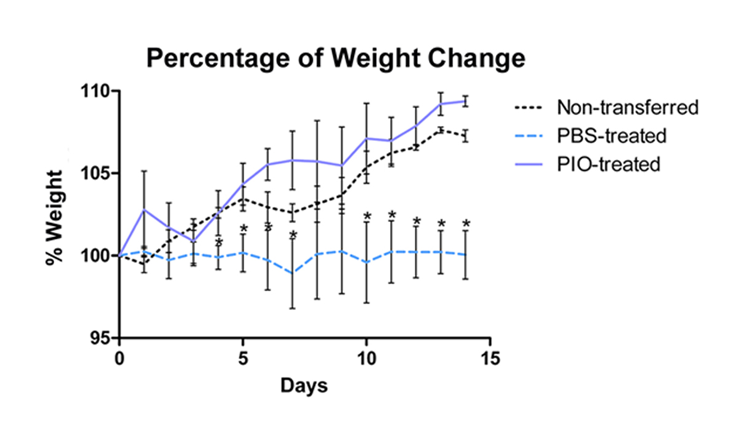

Supplement: Figure S11 — Improvement in mouse body weight following oral treatment with pioglitazone in RAG2-/- mice. RAG2-/- adoptive transfer recipient mice were treated with either PIO or PBS (control group) for 14 days and the average daily loss in body weights throughout the 14 day treatment was calculated. Data are represented as mean ± standard error. Points with an asterisk are significantly different when compared to the PBS-treated group (P<0.05). (TIF) [file pcbi.1003027.s011.tif]

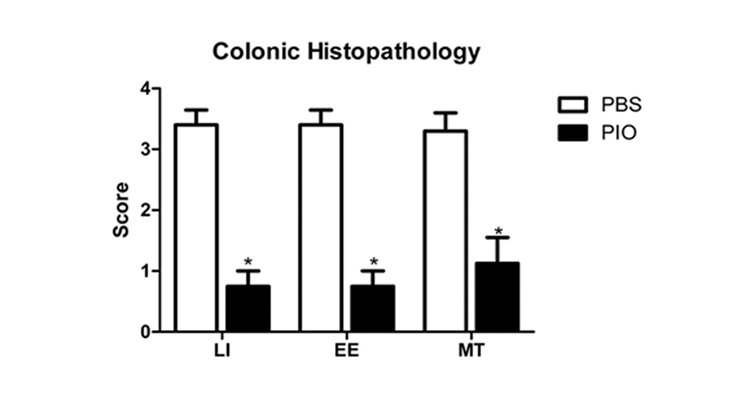

Supplement: Figure S12 — Histopathological analysis on colonic tissue from adoptive transfer studies. RAG2-/- adoptive transfer recipient mice were treated with either PIO or PBS (control group) for 14 days and histopathological assessment was performed. All specimens underwent blinded histological examination and were scored (0–4) on leukocyte infiltration (LI), epithelial erosion (EE) and mucosal wall thickening (MT) on day 14 after treatment. Data are represented as mean ± standard error. Points with an asterisk are significantly different at a given time point (P<0.05). (TIF) [file pcbi.1003027.s012.tif]

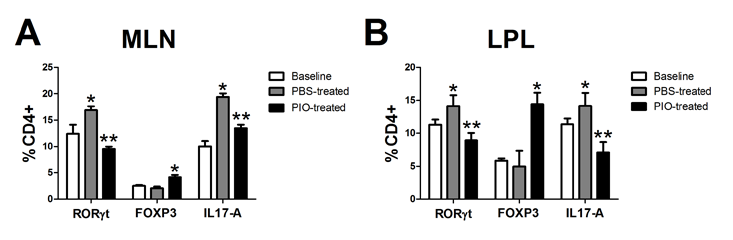

Supplement: Figure S13 — Pharmacological activation of peroxisome proliferator-activated receptor γ (PPARγ) favors a switch of Th17 cells towards an iTreg phenotype in vivo . RAG2-/- mice with induced chronic colitis were treated with either PBS or PIO for 14 days and flow cytometry were assessed at day 0 (baseline) and at the end of the treatment. (A) Accumulation of iTreg and Th17 cells in the mesenteric lymph nodes (MLN) (B) Accumulation of iTreg and Th17 cells in the colonic lamina propria (LP) of recipient mice. Data are represented as mean ± standard error. Points with an asterisk are significantly different at a given time point (P<0.05). (TIF) [file pcbi.1003027.s013.tif]
