# Supplementary material for: Systems Modeling of Molecular Mechanisms Controlling Cytokine-driven CD4+ T Cell Differentiation and Phenotype Plasticity
Source: PLoS Comput Biol. 2013 Apr 4;9(4):e1003027. doi: 10.1371/journal.pcbi.1003027 (PMC3617204; doi:10.1371/journal.pcbi.1003027)
Supplement: Figure S3 — Ordinary Differential Equations (ODE) triggering activation and inhibition regulatory and effector pathways in our CD4+ T cell model. Briefly, mass action and the Hill functions were used to reproduce CD4+ T cell behaviors in silico based on initial stimulation by external cytokines. (PDF) [file pcbi.1003027.s003.pdf]

$$\frac{d(eIFNg)}{dt} = (v_{IFNgpool} \cdot (\frac{[IFNgpool]^n}{[IFNgpool]^n + [eIFNg]^n + 0.001} - k_{IFNgpool} \cdot [eIFNg])) - (vf \cdot [eIFNg] \cdot [IFNgR] \cdot \frac{k^n}{[SOCS1-JAKs]^n + k^n} - Vr \cdot [IFNg - IFNgR]) + (k1 \cdot [IFNg])$$

$$\frac{d(eIL12)}{dt} = (v_{IL12pool} \cdot (\frac{[IL12pool]^n}{[IL12pool]^n + [eIL12]^n + 0.001} - k_{IL12pool} \cdot [eIL12])) - (vf \cdot [eIL12] \cdot [IL12R] \cdot \frac{k1^n}{[STAT6-P]^n + k1^n} \cdot \frac{k2^n}{[L-PPARg]^n + k2^n} - Vr \cdot [IL12 - IL12R]) + (k1 \cdot [IL12])$$

$$\frac{d(eIL21)}{dt} = (v_{IL21pool} \cdot (\frac{[IL21pool]^n}{[IL21pool]^n + [eIL21]^n + 0.001} - k_{IL21pool} \cdot [eIL21])) - (vf \cdot [eIL21] \cdot [IL21R] \cdot \frac{k^n}{[STAT5-P]^n + k^n} \cdot (1 + \frac{[IL17-IL17R]^n}{[IL17-IL17R]^n + k1^n} - Vr \cdot [IL21 - IL21R]) + (k1 \cdot [IL21])$$

$$\frac{d(eIL23)}{dt} = (v_{IL23pool} \cdot \left(\frac{[IL23pool]^n}{[IL23pool]^n + [eIL23]^n + 0.001} - k_{IL23pool} \cdot [eIL23]\right) - ((k1 \cdot [eIL23] - k2 \cdot [p40p19dimer]))$$

$$\frac{d(eIL4)}{dt} = (v_{IL4pool} \cdot (\frac{[IL4pool]^n}{[IL4pool]^n + [eIL4]^n + 0.001} - k_{IL4pool} \cdot [eIL4])) - (vf \cdot [eIL4] \cdot [IL4R] \cdot \frac{k^n}{[SOCS1-JAKs]^n + k^n} - Vr \cdot [IL4 - IL4R]) + (k1 \cdot [IL4])$$

$$\frac{d(eTGFb)}{dt} = (v_{TGFbpool} \cdot (\frac{[TGFbpool]^n}{[TGFbpool]^n + [eTGFb]^n + 0.001} - k_{TGFbpool} \cdot [eTGFb])) - (k1 \cdot [eTGFb] \cdot [TGFbR] - k2 \cdot [TGFb - TGFbR]) + (k1 \cdot [TGFb])$$

$$\frac{d(eIL2)}{dt} = (v_{IL2pool} \cdot (\frac{[IL2pool]^n}{[IL2pool]^n + [eIL2]^n + 0.001} - k_{IL2pool} \cdot [eIL2])) - (k1 \cdot [eIL2] \cdot [IL2R] - k2 \cdot [IL2 - IL2R]) + (k1 \cdot [IL2])$$

$$\frac{d(eIL6)}{dt} = (v_{IL6pool} \cdot (\frac{[IL6pool]^n}{[IL6pool]^n + [eIL6]^n + 0.001} - k_{IL6pool} \cdot [eIL6])) - (k1 \cdot [eIL6] \cdot [IL6R] - k2 \cdot [IL6 - IL6R]) + (k1 \cdot [IL6])$$

$$\frac{d(eIL17)}{dt} = (v_{IL17pool} \cdot (\frac{[IL17pool]^n}{[IL17pool]^n + [eIL17]^n + 0.001} - k_{IL17pool} \cdot [eIL17])) - (k1 \cdot [eIL17] \cdot [IL17R] - k2 \cdot [IL17 - IL17R]) + (k1 \cdot [IL17])$$

$$\frac{d(eIL10)}{dt} = (v_{IL10pool} \cdot (\frac{[IL10pool]^n}{[IL10pool]^n + [eIL10]^n + 0.001} - k_{IL10pool} \cdot [eIL10])) - (k1 \cdot [eIL10] \cdot [IL10R] - k2 \cdot [IL10 - IL10R]) + (k1 \cdot [IL10])$$

$$\frac{d(eIL18)}{dt} = (v_{IL18pool} \cdot (\frac{[IL18pool]^n}{[IL18pool]^n + [eIL18]^n + 0.001} - k_{IL18pool} \cdot [eIL18])) - (vf \cdot [eIL18] \cdot [IL18R] \cdot \frac{k^n}{[STAT6-P]^n + k^n} - vr \cdot [IL18 - IL18R]) + (k1 \cdot [IL18])$$

$$\frac{d(IL6-IL6R)}{dt} = (k1 \cdot [IL6] \cdot [IL6R] - k2 \cdot [IL6 - IL6R])$$

$$\frac{d(IL6R)}{dt} = -(k1 \cdot [eIL6] \cdot [IL6R] - k2 \cdot [IL6 - IL6R])$$

$$\frac{d(IL6)}{dt} = -k1 \cdot [IL6]$$

$$\frac{d(TGFb - TGFbR)}{dt} = (k1 \cdot [TGFb] \cdot [TGFbR] - k2 \cdot [TGFb - TGFbR])$$

$$\frac{d(TGFbR)}{dt} = -(k1 \cdot [eTGFb] \cdot [TGFbR] - k2 \cdot [TGFb - TGFbR])$$

$$\frac{d(TGFb)}{dt} = -k1 \cdot [TGFb]$$

$$\frac{d(IL2 - IL2R)}{dt} = (k1 \cdot [IL2] \cdot [IL2R] - k2 \cdot [IL2 - IL2R])$$

$$\frac{d(IL2R)}{dt} = -(k1 \cdot [eIL2] \cdot [IL2R] - k2 \cdot [IL2 - IL2R])$$

$$\frac{d(IL2)}{dt} = -k1 \cdot [IL2]$$

$$\frac{d(IL4 - IL4R)}{dt} = vf \cdot [eIL4] \cdot [IL4R] \cdot \frac{k^n}{[SOCS1 - JAKs]^n + k^n} - vr \cdot [IL4 - IL4R]$$

$$\frac{d(IL4R)}{dt} = -(vf \cdot [eIL4] \cdot [IL4R] \cdot \frac{k^n}{[SOCS1 - JAKs]^n + k^n} - vr \cdot [IL4 - IL4R])$$

$$\frac{d(IL4)}{dt} = -k1 \cdot [IL4] + (vf \cdot [pIL4] \cdot \frac{k1^n}{[STAT1 - P]^n + k1^n} \cdot \frac{k2^n}{[anti - IL4]^n + k2^n} \cdot \left(\frac{[IL4]^n}{[IL4]^n + k3^n} + \frac{[GATA3 - P]^n}{[GATA3 - P]^n + k4^n} + \frac{[STAT5 - P]^n}{[STAT5 - P]^n + k5^n}\right) - vr \cdot [IL4])$$

$$\frac{d(IFNg - IFNgR)}{dt} = vf \cdot [eIFNg] \cdot [IFNgR] \cdot \frac{k^n}{[SOCS1 - JAKs]^n + k^n} - vr \cdot [IFNg - IFNgR]$$

$$\frac{d(IFNgR)}{dt} = -(vf \cdot [eIFNg] \cdot [IFNgR] \cdot \frac{k^n}{[SOCS1 - JAKs]^n + k^n} - vr \cdot [IFNg - IFNgR])$$

$$\frac{d(IL12 - IL12R)}{dt} = vf \cdot [eIL12] \cdot [IL12R] \cdot \frac{k1^n}{[STAT6 - P]^n + k1^n} \cdot \frac{k2^n}{[L - PPARg]^n + k2^n} - vr \cdot [IL12 - IL12R]$$

$$\frac{d(IL12R)}{dt} = -(vf \cdot [eIL12] \cdot [IL12R] \cdot \frac{k1^n}{[STAT6 - P]^n + k1^n} \cdot \frac{k2^n}{[L - PPARg]^n + k2^n} - vr \cdot [IL12 - IL12R])$$

$$\frac{d(IL12)}{dt} = -k1 \cdot [IL12]$$

$$\frac{d(IL18-IL18R)}{dt} = vf \cdot [eIL18] \cdot [IL18R] \cdot \frac{k^n}{[STAT6-P]^n + k^n} - vr \cdot [IL18-IL18R]$$

$$\frac{d(IL18R)}{dt} = -(vf \cdot [eIL18] \cdot [IL18R] \cdot \frac{k^n}{[STAT6-P]^n + k^n} - vr \cdot [IL18-IL18R])$$

$$\frac{d(IL18)}{dt} = -k1 \cdot [IL18]$$

$$\frac{d(IL21-IL21R)}{dt} = vf \cdot [eIL18] \cdot [IL18R] \cdot \frac{k^n}{[STAT5-P]^n + k^n} \cdot (1 + \frac{[IL17-IL17R]^n}{[IL17-IL17R]^n + k1^n} - vr \cdot [IL21-IL21R]$$

$$\frac{d(IL12R)}{dt} = -(vf \cdot [eIL18] \cdot [IL18R] \cdot \frac{k^n}{[STAT5-P]^n + k^n} \cdot (1 + \frac{[IL17-IL17R]^n}{[IL17-IL17R]^n + k1^n} - vr \cdot [IL21-IL21R])$$

$$\frac{d(IL21)}{dt} = -k1 \cdot [IL21] + (vf \cdot [pIL21] \cdot \left(1 + \frac{[STAT3-P]^n}{[STAT3-P]^n + k^n}\right) - vr \cdot [IL21])$$

$$\frac{d(IL23R)}{dt} = -vf \cdot [p40p19dimer] \cdot [IL23R] \cdot \frac{k^n}{[Tbet-P]^n + k^n} - vr \cdot [IL23-IL23R]$$

$$\frac{d(IL23-IL23R)}{dt} = vf \cdot [p40p19dimer] \cdot [IL23R] \cdot \frac{k^n}{[Tbet-P]^n + k^n} - vr \cdot [IL23-IL23R]$$

$$\frac{d(IL10R)}{dt} = -(k1 \cdot [eIL10] \cdot [IL10R] - k2 \cdot [IL10-IL10R])$$

$$\frac{d(IL10-IL10R)}{dt} = k1 \cdot [eIL10] \cdot [IL10R] - k2 \cdot [IL10-IL10R]$$

$$\frac{d(IRAK1-P)}{dt} = vf \cdot [IRAK1] \cdot \left(1 + \frac{[IL18-IL18R]^n}{[IL18-IL18R]^n + k^n}\right) - vr \cdot [IRAK1-P])$$

$$\frac{\mathrm{d(p50p65dimer)}}{\mathrm{dt}} = \mathrm{vf}\cdot[\mathrm{p50}]\cdot[\mathrm{p65}]\cdot\frac{\mathrm{k1}^n}{[\text{L-PPARg}]^n+\mathrm{k1}^n}\cdot\frac{\mathrm{k2}^n}{[\mathrm{aFOXP3}]^n+\mathrm{k2}^n}\cdot\left(1+\frac{[\text{IRAK1-P}]^n}{[\text{IRAK1-P}]^n+\mathrm{k3}^n}\right)\text{-vr}\cdot[\mathrm{p50p65dimer}])$$

$$\frac{d(STAT4-P)}{dt} = vf \cdot [STAT4] \cdot \frac{k1^n}{[IL21-IL21R]^n + k1^n} \cdot \frac{k2^n}{[GATA3-P]^n + k2^n} \cdot \left(\frac{[IL12-IL12R]^n}{[IL12-IL12R]^n + k3^n} + \frac{[IL23-IL23R]^n}{[IL23-IL23R]^n + k4^n}\right) - vr \cdot [STAT4-P])$$

$$\frac{d(IFNg)}{dt} = -k1 \cdot [IFNg] + vf \cdot [pIFNg] \cdot \frac{k1^n}{[anti-IFNg]^n + k1^n} \cdot \frac{k2^n}{[STAT3-P]^n + k2^n} \cdot \frac{k3^n}{[L-PPARg]^n + k3^n} \cdot \left(\frac{[STAT4-P]^n}{[STAT4-P]^n + k4^n} + \frac{[p50p65dimer]^n}{[p50p65dimer]^n + k5^n} + \frac{[Tbet-P]^n}{[Tbet-P]^n + k6^n}\right) - vr \cdot [IFNg])$$

$$\frac{d(JAK1-P)}{dt} = vf \cdot [JAK1] \cdot \frac{k1^n}{[L-PPARg]^n + k1^n} \cdot \frac{k2^n}{[SOCS1-JAKs]^n + k2^n} \cdot \left(\frac{[IFNg-IFNgR]^n}{[IFNg-IFNgR]^n + k3^n}\right) - vr \cdot [JAK1-P])$$

$$\frac{d(STAT1-P)}{dt} = vf \cdot [STAT1] \cdot \frac{k^n}{[L-PPARg]^n + k^n} \cdot \left(\frac{[IL21-IL21R]^n}{[IL21-IL21R]^n + k1^n} + \frac{[IFNg-IFNgR]^n}{[IFNg-IFNgR]^n + k2^n} + \frac{[JAK1-P]^n}{[JAK1-P]^n + k3^n}\right) - vr \cdot [STAT1-P])$$

$$\frac{d(p40p19dimer)}{dt} = k1 \cdot [eIL23] - k2 \cdot [p40p19dimer] - \left(vf \cdot [p40p19dimer] \cdot [IL23R] \cdot \frac{k^n}{[Tbet-P]^n + k^n} - vr \cdot [IL23-IL23R]\right) + vf \cdot [p40] \cdot [p19] \cdot \frac{k^n}{[L-PPARg]^n + k^n} \cdot \left(1 + \frac{[IL6-IL6R]^n}{[IL6-IL6R]^n + k1^n}\right) - vr \cdot [p40p19dimer])$$

$$\frac{d(IL17)}{dt} = -k1 \cdot [IL17] + \left(vf \cdot [pIL17] \cdot \frac{k^n}{[L-PPARg]^n + k^n} \cdot (\frac{[IL23-IL23R]^n}{[IL23-IL23R]^n + k1^n} + \frac{[RORgtL]^n}{[RORgtL]^n + k2^n} + \frac{[STAT4-P]^n}{[STAT4-P]^n + k3^n} - vr \cdot [IL17]\right) + (v_{IL17pool} \cdot (\frac{[IL17pool]^n}{[IL17pool]^n + [IL17]^n + 0.001} - k_{IL17pool} \cdot [IL17]))$$

$$\frac{d(IL10)}{dt} = -k1 \cdot [IL10] + vf \cdot [pIL10] \cdot \frac{k^n}{[IL23-IL23R]^n + k^n} \cdot \left(\frac{[L-PPARg]^n}{[L-PPARg]^n + k1^n} + \frac{[IL21-IL21R]^n}{[IL21-IL21R]^n + k2^n} + \frac{[GATA3-P]^n}{[GATA3-P]^n + k3^n} + \frac{[STAT5-P]^n}{[STAT5-P]^n + k4^n}\right) - vr \cdot [IL10])$$

$$\frac{d(RORgtL)}{dt} = vf \cdot [RORgt] \cdot \frac{k1^n}{[aFOXP3]^n + k1^n} \cdot \frac{k2^n}{[L-PPARg]^n + k2^n} \cdot \left(\frac{[STAT3-P]^n}{[STAT3-P]^n + k3^n} + \frac{[TGFb-TGFbR]^n}{[TGFb-TGFbR]^n + k4^n}\right) - vr \cdot [RORgtL])$$

$$\frac{d(STAT3-P)}{dt} = vf \cdot [STAT3] \cdot \frac{k^n}{[L-PPARg]^n + k^n} \cdot \left(\frac{[IL21-IL21R]^n}{[IL21-IL21R]^n + k1^n} + \frac{[IL23-IL23R]^n}{[IL23-IL23R]^n + k2^n} + \frac{[IL10-IL10R]^n}{[IL10-IL10R]^n + k3^n} + \frac{[IL6-IL6R]^n}{[IL6-IL6R]^n + k4^n}\right) - vr \cdot [STAT3-P])$$

$$\frac{d(STAT5-P)}{dt} = vf \cdot [STAT5] \cdot \left(1 + \frac{[IL2-IL2R]^n}{[IL2-IL2R]^n + k^n}\right) - vr \cdot [STAT5-P])$$

$$\frac{d(SOCS1-JAKs)}{dt} = vf \cdot [SOCS1] \cdot \left(\frac{[STAT1-P]^n}{[STAT1-P]^n + k1^n} \cdot \frac{[Tbet-P]^n}{[Tbet-P]^n + k2^n}\right) - vr \cdot [SOCS1-JAKs])$$

$$\frac{d(Tbet-P)}{dt} = vf \cdot [Tbet] \cdot \frac{k1^n}{[TGFb-TGFbR]^n + k1^n} \cdot \frac{k2^n}{[STAT3-P]^n + k2^n} \cdot \left(\frac{[STAT4-P]^n}{[STAT4-P]^n + k3^n} + \frac{[STAT1-P]^n}{[STAT1-P]^n + k4^n} + \frac{[Tbet-P]^n}{[Tbet-P]^n + k5^n}\right) - vr \cdot [Tbet-P])$$

$$\frac{d(GATA3-P)}{dt} = vf \cdot [GATA3] \cdot \frac{k1^n}{[GATA3-P]^n + k1^n} \cdot \frac{k2^n}{[STAT3-P]^n + k2^n} \cdot \frac{k3^n}{[TGFb-TGFbR]^n + k3^n} \cdot \left(\frac{[STAT6-P]^n}{[STAT6-P]^n + k4^n} + \frac{[L-PPARg]^n}{[L-PPARg]^n + k5^n}\right) - vr \cdot [GATA3-P])$$

$$\frac{d(STAT6-P)}{dt} = vf \cdot [STAT6] \cdot \frac{k^n}{[IFNg-IFNgR]^n + k^n} \cdot \frac{[IL4-IL4R]^n}{[IL4-IL4R]^n + k1^n} - vr \cdot [STAT6-P])$$

$$\frac{d(PPARg)}{dt} = -vf \cdot [PPARg] \cdot [Ligand] \cdot \frac{k^n}{[IFNg-IFNgR]^n + k^n} \cdot (1 + \frac{[IL4-IL4R]^n}{[IL4-IL4R]^n + k1^n}) - vr \cdot [L-PPARg])$$

$$\frac{d(L-PPARg)}{dt} = vf \cdot [PPARg] \cdot [Ligand] \cdot \frac{k^n}{[IFNg-IFNgR]^n + k^n} \cdot (1 + \frac{[IL4-IL4R]^n}{[IL4-IL4R]^n + k1^n}) - vr \cdot [L-PPARg])$$

$$\frac{d(aFOXP3)}{dt} = vf \cdot [FOXP3] \cdot \frac{k1^n}{[STAT6-P]^n + k1^n} \cdot \frac{k2^n}{[STAT3-P]^n + k2^n} \cdot \frac{k3^n}{[IL6-IL6R]^n + k3^n} \cdot \left(\frac{[TGFb-TGFbR]^n}{[TGFb-TGFbR]^n + k4^n} + \frac{[STAT1-P]^n}{[STAT1-P]^n + k5^n} + \frac{[STAT5-P]^n}{[STAT5-P]^n + k6^n} + \frac{[L-PPARg]^n}{[L-PPARg]^n + k7^n}\right) - vr \cdot [aFOXP3])$$

$$\frac{d(IL17R)}{dt} = -(k1 \cdot [eIL17] \cdot [IL17R] - k2 \cdot [IL17-IL17R])$$

$$\frac{d(IL17-IL17R)}{dt} = k1 \cdot [eIL17] \cdot [IL17R] - k2 \cdot [IL17-IL17R]$$

$$FOXP3R = \frac{[aFOXP3]}{[BActin].InitialValue}$$

$$IL17R = \frac{[IL17]}{[BActin].InitialValue}$$
