# Supplementary material for: Systems Modeling of Molecular Mechanisms Controlling Cytokine-driven CD4+ T Cell Differentiation and Phenotype Plasticity
Source: PLoS Comput Biol. 2013 Apr 4;9(4):e1003027. doi: 10.1371/journal.pcbi.1003027 (PMC3617204; doi:10.1371/journal.pcbi.1003027)
Supplement: Text S1 — Basic information on model creation, model calibration and simulation process. Briefly, the model was constructed using Th1, Th2, Th17 and iTreg information from the literature. Parameter estimation was ran using the Complex Pathway Simulator (COPASI) and quality control was performed to ensure proper initialization and fate. Afterwards, in silico experimentation was run to produce computational hypotheses. (DOCX) [file pcbi.1003027.s021.docx]

Supplementary Materials for

**Systems modeling of molecular mechanisms controlling cytokine-driven CD4+ T cell differentiation and phenotype plasticity**

Adria Carbo, Raquel Hontecillas, Barbara Kronsteiner, Monica Viladomiu, Mireia Pedragosa, Pinyi Lu, Casandra W Philipson, Stefan Hoops, Madhav Marathe, Stephen Eubank, Keith Bisset, Katherine Wendelsdorf, Abdul Jarrah, Yongguo Mei, and Josep Bassaganya-Riera^*^

*To whom correspondence should be addressed. E-mail: jbassaga@vt.edu

**Section 1: Model creation**

The population of CD4+ T cells is functionally and phenotypically heterogeneous consisting of at least four subsets involved in coordinating various aspects of adaptive immunity. Upon antigenic stimulation by antigen-presenting cells, naïve CD4+ T cells (Th0) expand and differentiate into at least three effector cell subsets referred to as Th1, Th2, Th17, and induced regulatory T (iTreg) cells (Figure S1). In addition to the four Th phenotypes studied and modeled in this project other CD4+ T cell phenotypes have been characterized, including transforming growing factor β (TGF-β)-producing CD4+ T cells (Th3) [[1](#_ENREF_1)], IL-10-producing CD4+ T cells (Tr1) [[2](#_ENREF_2)], IL-9-producing CD4+ T cells (Th9) [[3](#_ENREF_3)] and T follicular helper (Tfh) cells located in the follicular regions of lymph nodes and spleen [[4](#_ENREF_4),[5](#_ENREF_5)]. Signaling pathways controlling fates on these phenotypes are closely connected to the four core subsets (Th1, Th2, Th17 and iTreg). Each of these phenotypes is characterized by distinct effector and regulatory functions, which are regulated by a signature pattern of cytokines and multiple transcription factors. The signaling pathways that lead to these four predominant fates are cross-regulated via feedback loops that facilitate a balanced immune response to pathogens or abnormal cells while avoiding chronic inflammation and autoimmunity.

We present for the first time a mathematical and computational model built upon the current paradigms of molecular interactions that occur in CD4+ T cells. This model will help us to elucidate the regulatory mechanisms underlying CD4+ T cell differentiation, identify novel putative CD4+ T cell subsets, and study the dynamics of Th cell differentiation. Previous modeling efforts have also focused on the CD4+ T cell. For instance, Mendoza reported a logical network model for controlling the differentiation process in CD4+ T cells [[6](#_ENREF_6)], however, that model was build upon the Th1 versus Th2 paradigm, without considering Th17 or iTreg subsets. Additional models of immunity are available for predicting the generation of memory cells [[7](#_ENREF_7),[8](#_ENREF_8)] and determining the role of IL-2 in the interplay between effector and regulatory phenotypes [[9](#_ENREF_9)]. There is also a comprehensive review on differentiation of effector CD4+ T cell populations by Zhu and colleagues [[10](#_ENREF_10)]. Recent publications also reported on modeling approaches for specific CD4+ T cell phenotypes, such as the regulation of Th1 by T-bet, IL-12 and interferon-γ (IFN-γ) [[11](#_ENREF_11)] or the regulation of the crosstalk between Th17 and iTreg by quantifying the master regulators [[12](#_ENREF_12)]. Other studies have focused on the interaction between more than two phenotypes using logical models [[13](#_ENREF_13)]. However, our extended ODE-based model is the first to illustrate in a detailed and comprehensive manner the intracellular regulatory networks controlling fate determination for all four phenotypes in a deterministic way (i.e., Th1, Th2, Th17 and iTreg). Specifically, we have extended previous models by adding some new detailed interactions for the Th1/Th2-related pathways, including new pathways controlling plasticity between Th17 and iTreg cells, as well as the crosstalk among these pathways. In addition, in contrast to previous studies and given the initial results of the sensitivity analysis, our structural network model includes the modulation of this process by the nuclear receptor peroxisome proliferator-activated receptor gamma (PPARγ).

Three distinct signals regulate CD4+ T cell activation and differentiation: a signal from the T cell receptor (TCR) interacting with MHC, a co-stimulatory signal (i.e., CD28 interacting with B7.1 or B7.2 on antigen presenting cells), and a cytokine-driven signal. Other studies have focused on CD4+ T cell proliferation [[14](#_ENREF_14)], TCR signaling [[12](#_ENREF_12)] or co-stimulatory signals [[15](#_ENREF_15)]. In this report, we assemble the knowledge about non-cognate interactions controlling the CD4+ T cell differentiation process (i.e., cytokine milieu, signaling pathways and transcription factors) available in the literature into a comprehensive network model. This is a first step toward a more comprehensive understanding of the dynamics of the CD4+ T cell differentiation process at the systems level. Thus, we are describing activation pathways by phenotype, as well as the inhibitory mechanisms that lead to the induction or suppression of a CD4+ T cell phenotype.

**T helper 1 cells**

A naïve T cell can differentiate into a Th1 phenotype through two major signaling pathways which have recently been shown to be interconnected and their expression is coordinated by antigen-induced signaling [[11](#_ENREF_11),[16](#_ENREF_16),[17](#_ENREF_17)]. The first pathway involves antigen recognition through the T cell receptor (TCR) that activates the signaling pathway of IFN-γ, and the transcription factors signal transducer of activation of transcription (STAT)-1 and T-bet. IFN- γ binds to its receptor IFN-γR, on the T cell surface, and activates janus kinase-1 (JAK-1) and STAT-1 [[18-20](#_ENREF_18)] which leads to the expression of T-bet in Th1 [[21](#_ENREF_21),[22](#_ENREF_22)]. Furthermore, T-bet can induce its own transcription [[23](#_ENREF_23)] and is known to induce IFN-γ expression [[24](#_ENREF_24)], thereby creating a positive feedback loop. Independent of IFN-γ a sustained expression of T-bet in human Th2 cells induces Th1 cytokines and represses Th2 cytokines [[24](#_ENREF_24),[25](#_ENREF_25)]. T-bet is also capable of activating suppressor of cytokine signaling (SOCS)-1, which then blocks IL-4R signaling in response to IL-4 stimulation [[26](#_ENREF_26)], therefore inhibiting the Th2 phenotype and favoring Th1 differentiation. In addition to T-bet, another strong activator of SOCS-1 is STAT-1 which also favors Th1 differentiation [[27](#_ENREF_27)]. On the other hand, SOCS-1 can inhibit JAK-1 and block the activation of STAT-1 by IFN-γ *in vivo* [[28](#_ENREF_28),[29](#_ENREF_29)], thereby representing a negative feedback loop that could suppress Th1 differentiation.

The other major pathway for a naïve CD4+ T cell to differentiate into a Th1 phenotype involves the IL-12/STAT-4 axis [[24](#_ENREF_24),[30](#_ENREF_30)] by the activation of STAT-4 through the signaling of antigen-presenting cell (APC)-derived IL-12 [[23](#_ENREF_23),[31](#_ENREF_31)], where STAT-4 up-regulates IFN-γ expression [[32](#_ENREF_32)]. Furthermore, STAT-4 is not only capable of inducing Th1 differentiation independently of T-bet, but it is also essential for Th1 differentiation in the absence of T-bet [[30](#_ENREF_30)]. Indeed, Furuta et al. (2008) showed that Th1 differentiation was severely impaired in both T-bet^–/–^ CD4^+^ T cells and STAT4^–/–^ CD4^+^ T cells, which suggests that STAT-4 activates T-bet directly or indirectly [[30](#_ENREF_30)].

Although IL-18 is not required for the development of Th1 cells, it is essential for the effective induction and activation of Th1 cells by IL-12 [[33](#_ENREF_33)], as it synergizes with IL-12 in the induction of IFN-γ by activating STAT-4 and promoting IFN-γ activation [[34](#_ENREF_34)]. IL-18 signals through the IL-1 receptor associated kinase (IRAK-1) to induce the accumulation of NF-κB[[35](#_ENREF_35)] which then leads to the induction of IFN-γ[[36](#_ENREF_36)]. Indeed, nuclear factor-kappaB (NF-κB) and STAT-4 synergize to induce IFN-γ [[37](#_ENREF_37),[38](#_ENREF_38)].

Next we describe the underlying mechanisms that inhibit naïve T cells from differentiating into a Th1 phenotype. There is evidence that STAT-6 inhibits the IL-12/STAT-4 pathway [[39](#_ENREF_39)] and is required for the down-regulation of IL-18Rα [[40](#_ENREF_40)]. The over-expression of STAT-3 reduces the expression of the trans-acting T cell-specific factor (GATA-3), a transcription factor involved in Th2 differentiation, and T-bet, and hence inhibits the differentiation into Th1 and Th2 [[41](#_ENREF_41)]. The transcription factor forkhead box P3 (FOXP3), a marker for Treg cells, inhibits the production of IFN-γ by physically binding to and blocking NF-κB from inducing IFN-γ [[42](#_ENREF_42)]. PPARγ ligands can directly decrease IFN-γ expression [[43](#_ENREF_43)]. At the same time, however, the inactivation of STAT-3 by PPARγ [[44](#_ENREF_44)] could activate IFN-γ expression as STAT-3 inhibits IFN-γ. In macrophages, PPARγ down-regulates the expression of pro-inflammatory cytokines by antagonizing the activities of transcription factors such as activator protein (AP)-1, STAT and NF-κB [[45](#_ENREF_45)], and in epithelial cells it favors the nucleocytoplasmic shuttling of the activated p65 subunit of NF-κB [[46](#_ENREF_46)]. It remains unknown whether these mechanisms observed in macrophages and epithelial cells play a role in CD4+ T cell differentiation.

**T helper 2 cells**

Naïve T helper cells will differentiate into the effector Th2 phenotype, characterized by the expression of IL-4, IL-5 and IL-13, through two apparently independent pathways, namely, the IL-4/STAT-6 and IL-2/STAT-5 axis [[47](#_ENREF_47)]. GATA-3 is the common link between both pathways[[47](#_ENREF_47)]. Binding of IL-4 to its receptor leads to the phosphorylation of STAT-6 which induces GATA-3 expression[[48](#_ENREF_48)]. GATA-3 is known to activate IL-4[[49](#_ENREF_49)], which creates a positive feedback loop ensuring the stability of Th2 fate.

Enhanced IL-2 signaling by binding to its receptor and inducing STAT-5 is an essential pathway for Th2 differentiation [[50](#_ENREF_50),[51](#_ENREF_51)]. Neutralization of IL-2 abolishes early IL-4 production without affecting early GATA-3 expression [[52](#_ENREF_52)], which suggests alternative mechanisms for activating GATA-3. Experimental results in mice indicate that GATA-3 is capable of inducing its own expression [[49](#_ENREF_49)]. Recently it has been reported that Notch directly regulates GATA-3 expression, and synergistically contributes to Th2 differentiation [[53](#_ENREF_53),[54](#_ENREF_54)]. These findings may explain Th2 differentiation *in vivo* without the stimulation by IL-4.

PPARγ expression in activated T cells is dependent on IL-4 [[55](#_ENREF_55)], indicating a link with the Th2 fate. Direct physical interactions between PPARγ and NFAT can result in inhibition of IL-2 production by CD4+ T cells [[56](#_ENREF_56)]. While IL-4 upregulates PPARγ expression, treatment of CD4^+^ T cells with PPARγ agonists (i.e., ciglitazone or 15dPGJ2) triggered the physical association between PPARγ and NFATc1, resulting in IL-4 promoter inhibition and decreased IL-4 production [[57](#_ENREF_57)], suggesting the existence of a regulatory mechanism that prevents excessive differentiation towards the Th2 phenotype. Also, 13-hydroxyoctadecadienoic acid, an endogenously generated PPARγ agonist, down-regulated IL-2 production by human peripheral blood T lymphocytes by reducing NFAT and NF-κB binding to the IL-2 promoter [[58](#_ENREF_58)]. Moreover, IL-4 was shown to simultaneously increase the expression of PPARγ and 12-15-lipooxygenase, the enzyme involved in the generation of 13-hydroxyoctadecadienoic acid [[59](#_ENREF_59)]. Thus, it has been proposed that IL-4 indirectly down-regulates IL-2 production by T cells through a PPARγ -dependent mechanism [[58](#_ENREF_58),[59](#_ENREF_59)].

The differentiation into Th2 could possibly be inhibited through a variety of mechanisms that have also been incorporated in our network model. For instance, the over-expression of SOCS-1 in Th2 cells represses STAT-6 activation and profoundly inhibits IL-4-induced proliferation [[60](#_ENREF_60)] and SOCS-1 inhibits IL-4R from phosphorylating STAT-6[[61](#_ENREF_61),[62](#_ENREF_62)]. Furthermore, STAT-1 is required for the repression of IL-4-induced gene expression by IFN-γ [[63](#_ENREF_63)]. Also, IFN-γ was shown to inhibit STAT-6 by suppressing its phosphorylation by IL-4R[[64](#_ENREF_64)]. On the other hand, the iTreg cell-derived cytokine transforming growth factor-β (TGF-β) inhibits GATA-3 expression at the transcriptional level, however, it does not interfere with IL-4 signaling [[65](#_ENREF_65)]. FOXP3 interacts with NFAT, such that NFAT becomes unable to induce IL-4 expression [[42](#_ENREF_42)], thereby rearing it unable to activate T cells in response to antigenic stimulation via the TCR [[66](#_ENREF_66)].

**T helper 17 cells**

Th17 cells are characterized by their production of the cytokine IL-17. TGF-β, together with pro-inflammatory cytokines IL-6 or IL-21 and IL-23, orchestrate the differentiation of CD4+ T cells into the Th17 phenotype in a concentration-dependent manner [[67](#_ENREF_67),[68](#_ENREF_68)]. It has been demonstrated that TGF-β synergizes with IL-6 [[69](#_ENREF_69)] or IL-21 [[70](#_ENREF_70),[71](#_ENREF_71)] to promote the expression of IL-17. This is achieved through stimulation of retinoid-related orphan receptor (ROR)γt by IL-6 through the transcription of STAT-3 [[72](#_ENREF_72),[73](#_ENREF_73)], which in turn induces expression of IL-17 [[41](#_ENREF_41),[74](#_ENREF_74)]. While RORγt is essential for the differentiation of naïve CD4+ T cells into Th17 effector cells, IL-23 is required for maintaining and stabilizing the Th17 phenotype[[75](#_ENREF_75)], and it acts through the IL-23R [[76](#_ENREF_76)].

The Th17 differentiation process is very similar in mice and humans [[77](#_ENREF_77),[78](#_ENREF_78)]. As in mice, TGF-β, IL-23 and pro-inflammatory cytokines (IL-1β and IL-6) were all essential for human Th17 differentiation [[79](#_ENREF_79)]. In this regard, TGF-β along with IL-21 and IL-23 stimulate the expression of RORγt, which in turn induces expression of IL-17 [[41](#_ENREF_41),[56](#_ENREF_56)]. Th17 cells also secrete IL-21 [[77](#_ENREF_77)]. IL-6, IL-21 but not TGF-β induced IL-23 receptor up-regulation in stimulated naive CD4+ T cells[[56](#_ENREF_56)].

The differentiation of Th17 cells is antagonized by transcription factors that control the differentiation of other lineages, such as T-bet (Th1), GATA-3 (Th2), and FOXP3 (Treg) [[80](#_ENREF_80)]. T-bet inhibits IL-23 and hence is critical for the stability of the Th17 phenotype[[81](#_ENREF_81),[82](#_ENREF_82)]. GATA-3 acts as an inhibitor of Th17 [[83](#_ENREF_83)], this could be mediated by the inhibition of STAT-4, a promoter of IL-17 expression. FOXP3 inhibits the RORγt-driven transcription of IL-17 by directly suppressing RORγt [[68](#_ENREF_68),[84](#_ENREF_84)]. Furthermore, the IL-2/STAT-5 axis constrains Th17 [[78](#_ENREF_78)] in part through a FOXP3-dependent mechanism, since STAT-5 activates FOXP3 [[85](#_ENREF_85)] as well as through the inhibition of the STAT-3/IL-21 pathway [[86](#_ENREF_86)]. Double positive FOXP3 RORγt T-helper cells have been identified as an intermediary that displays suppressive function [[87](#_ENREF_87)]. Of note, the equilibrium of this double positive balance coexist and it is tightly controlled, suggesting that a perturbed equilibrium coming from a change in cytokine milieu might lead to a skewed phenotype [[88](#_ENREF_88)]. In line with this fact, IL-2 signaling via STAT-5 constrains Th17 generation [[89](#_ENREF_89)] and IL-2 has been found to regulate the development of Th17 via FOXP3+ regulatory T cells[[90](#_ENREF_90)].

Another known inhibitor of Th17 differentiation is PPARγ as its activation can inhibit STAT-3 and hence contribute to the down-regulation of IL-17 through the IL-6/STAT-3/RORγt/IL-17 axis [[91](#_ENREF_91),[92](#_ENREF_92)]. Although TGF-β alone is not capable of inducing IL-17 and hence producing Th17, it is necessary for differentiation into Th17 and its absence induced a shift from a Th17 profile to a Th1-like profile [[77](#_ENREF_77),[79](#_ENREF_79)]. Moreover, PPARγ is a key negative regulator of human and mouse Th17 differentiation since it reduced RORγt transcription on a single-cell level [[93](#_ENREF_93)].

**Regulatory CD4+ T cells**

Induced or adaptive Treg (iTreg) cells can be generated from naïve CD4+ T cells by the stimulation of TCR and in the presence of TGF-β1 [[94](#_ENREF_94),[95](#_ENREF_95)] and the absence of IL-6 [[69](#_ENREF_69)]. TGF-β induces the expression of FOXP3, which is the master regulator for Treg [[95-97](#_ENREF_95)], and the IL-2/STAT-5 pathway is essential for the up-regulation of FOXP3 [[98](#_ENREF_98)]. The participation of TGF-β in the differentiation of Th17 cells places the Th17 lineage in close relationship with CD4^+^CD25^+^FOXP3^+^ iTregs, as TGF-β also induces differentiation of naive T cells into FOXP3^+^ iTregs in the peripheral immune compartment [[77](#_ENREF_77)]. The key difference that drives a TGF-β-stimulated CD4+ T cell towards Th17 or iTreg is the presence or absence of IL-6, respectively. Interestingly, iTreg cells can differentiate into pathogenic Th17 in the presence of IL-6 and/or IL-23 [[99](#_ENREF_99)], indicating plasticity in lineage commitment.

STAT-1 is also critical for the induction of iTreg cells. STAT1-deficient mice developed a functional impairment of iTreg cells [[100](#_ENREF_100),[101](#_ENREF_101)]. Recently, it was shown that FOXP3 expression is boosted by IFN-γ through the activation of STAT-1 which then directly binds to the FOXP3 promoter [[102](#_ENREF_102)].

PPARγ ligands enhance the differentiation of CD4+ T cells into iTreg cells [[103](#_ENREF_103),[104](#_ENREF_104)], although the underlying mechanisms are incompletely understood. Additionally, PPARγ ligands inhibit the production of pro-inflammatory cytokines, including IL-6 [[45](#_ENREF_45)]. In turn, IL-6 inhibits the expression of FOXP3 and hence favors Th17 over the iTreg phenotype [[96](#_ENREF_96),[105](#_ENREF_105)]. Thus, in the presence of PPARγ activation there is less IL-6 and a suppressed IL-6 mediated inhibition of FOXP3 that will favor the iTreg phenotype and facilitate anti-inflammatory responses and prevention of autoimmune disease.

Differentiation of CD4+ T cells into iTreg is inhibited through multiple mechanisms, including negative regulation of FOXP3 expression via GATA-3 [[106](#_ENREF_106)], IL-4-mediated inhibition of FOXP3 through STAT-6 [[107](#_ENREF_107)], and inhibition of TGF-β-induced FOXP3 by IL-6 and IL-21 [[70](#_ENREF_70)]. The latter mechanism of inhibition of iTreg differentiation appears to be mediated via STAT-3 activation, since IL-6 fails to inhibit FOXP3 in STAT-3-deficient mice [[108](#_ENREF_108)].

Interestingly, IFN-γ-deficient-mice had more FOXP3-positive cells than wild-type mice in all secondary lymphoid organs except the thymus [[109](#_ENREF_109)]. However, T-bet- or IL-4Rα-deficient mice did not show a similar increase. *In vitro* differentiation studies showed that conversion of naïve CD4+ T cells into FOXP3-positive iTreg cells by TGF-β was significantly inhibited by IFN-γ in a STAT-1-dependent manner. In an earlier study [[110](#_ENREF_110)], autocrine IFN-γ production regulated TGF-β-driven FOXP3 expression in iTreg and suppressed the conversion of naïve CD4+ T cells into FOXP3^+^ iTreg cells. However, IFN-γ is critically required for the conversion of naïve T cells to iTregs in a mouse model of multiple sclerosis [[101](#_ENREF_101)]. Furthermore, in human iTreg differentiation, a mechanism by which the STAT-1-activating cytokines IL-27 and IFN-γ amplify TGF-β-induced FOXP3 expression is revealed [[102](#_ENREF_102)]. Finally, recent reports show that the transcription factors for Th1, Th2, and Th17 cells, T-bet, GATA-3, and RORγt, respectively, can also be co-expressed in some Treg cells [[111-113](#_ENREF_111)], thereby indicating the existence of intermediate phenotypes. However, the molecular network leading to these intermediate phenotypes and their function remain largely unknown. The better understanding of the dynamics of iTreg differentiation is important for driving the informed development of possible Treg cell-based therapeutics against immune-mediated diseases.

**Importance of PPARγ in CD4+ T cell differentiation**

Inflammation is at the core of most human diseases, including chronic, infectious and immune-mediated. Activation of PPARγ, a widely expressed transcription factor, represents a conserved anti-inflammatory mechanism involved in the prevention of cancer [[114](#_ENREF_114),[115](#_ENREF_115)], diabetes [[116-118](#_ENREF_116)], atherosclerosis [[119](#_ENREF_119)], obesity [[120](#_ENREF_120)], infectious [[121-125](#_ENREF_121)] and immune-mediated diseases [[126-129](#_ENREF_126)]. Thus, modeling the mechanisms by which PPARγ regulates CD4+ T cell differentiation and function will facilitate a rational development of anti-inflammatory drugs and immunotherapeutics.

At the cellular level, iTreg express 10-fold greater amounts of PPARγ than Th1 cells [[130](#_ENREF_130)] and PPARγ is required for naturally occurring Treg-mediated protection from colitis [[129](#_ENREF_129)]. Moreover, PPARγ has been identified as a key down-regulator of differentiation of CD4+ T cells into Th17 [[131](#_ENREF_131)] a phenotype associated with inflammation. In macrophages, PPARγ favors a switch from a pro-inflammatory “classically activated” M1 to an M2 “alternatively activated” anti-inflammatory phenotype [[132](#_ENREF_132)]. Since PPARγ is ubiquitously expressed in the gut, tracing clinical improvements from therapeutic interventions with thiazolidinediones (TZD) and other PPARγ ligands back to concrete PPARγ-initiated immunological mechanisms has proven extremely challenging. PPARγ activity delineates the susceptibility to intestinal inflammation ranging from highly pro-inflammatory (low expression or activation) to anti-inflammatory (high expression or activation) states. We have developed a multiscale model of the intestine to understand how PPARγ modulates the immune response dynamics, gut pathology and anti-inflammatory responses [[133](#_ENREF_133)]. The initial level of granularity was cellular (immune and epithelial cells), with multiple tissues and compartments such as lumen, colonic lamina propria (LP) and mesenteric lymph nodes (MLN) [[133](#_ENREF_133)].

Here we present a higher resolution structural model network with molecular granularity that illustrates the principal pathways controlling the CD4+ T cell differentiation process towards Th1, Th2, Th17 and iTreg. An additional and novel feature of our model is that we describe the role of PPARγ as a central modulator of CD4+ T cell differentiation and function.

**Section 2: Modeling process**

Generating a mathematical model usually is comprised of three steps: first, a translation from the literature into a structural network is needed. The architecture of the model has to be assembled based on literature fates. Secondly, data extracted from the literature and data generated by our laboratory is inserted in the model to adjust the dynamics of the model and ensure the correct trends and behaviors of different molecules in the model. This process is known as ‘parameter estimation’. Once the parameters are set, a quality control check is needed to guarantee that signaling pathways are being activated promptly at the correct time with the right signal.

At this point, the model is ready to start running *in silico* experimentation and generating predictions with the right initializations. Ultimately, computational results will be generated, *in vitro* and *in vivo* validation studies are performed and the data generated in those studies is used to re-calibrate the model, using ‘parameter estimation’ again, thus closing and completing the modeling process. This iterative process is outlined in Figure S2.

**Parameter estimation for dynamics adjustment**

Once all the relationships between molecules were set, they were incorporated in the CellDesigner diagram representing a single CD4+ T cell (Figure 1). This diagram represents the cellular response of one CD4+ T cell activating and inhibiting reaction that take place in three different places: the extracellular environment, the cytoplasm and the nucleus space. Since CellDesigner [[134](#_ENREF_134)], a software package that enables users to describe molecular interactions using a well-defined and consistent graphical notation, and our MIEP-developed modeling software, the COmplex PAthway SImulator (COPASI) [[135](#_ENREF_135)] are Systems Biology Markup Language (SBML)-compliant an import was made into COPASI and the rate laws were adjusted to create the ordinary differential equations (Figure S3). To model CD4+ T cell differentiation, the hill function and mass action equations were used. While the Hill Coefficient allowed us to quantify the effect of a ligand binding a macromolecule through cooperative binding, mass action laws can represent dynamic equilibriums for elementary reactions, considering products as a proportion of the participating molecules in the reaction.

The parameter estimation computational approach was used to determine the unknown constants driving the dynamics of the model. Briefly, we used the Particle Swarm Optimization (PSO) [[136](#_ENREF_136)] algorithm to obtain computational values for our model parameters in order to fit our experimental data (Table S1) to the model. PSO is a global search algorithm and thus depends only minimally on the initial guess of each parameter and therefore avoids the subjective estimation caused by initial guesses in local methods as Levenberg-Marquardt. PSO has been used in other publications for the same purpose [[137](#_ENREF_137)].

Given the complexity of the model, the parameter estimation task was split into different sub-estimations that would run faster. Seven different parameter estimations were run successfully, including a parameter estimation for each phenotype (Th1, Th2, Th17 and iTreg), plus an extra one for PPARγ calibration, one for the Th1/Th2 crosstalk and a last one called ‘global parameter estimation’ that would include all these last six mentioned. Next, the ‘non-zero-gradient’ approach was performed. This step consists of assessing all the values with the gradients and check, value per value and parameter per parameter, which of those have the lowest or highest gradient. This approach can inform of which values have to be used for each phenotype and reaction. For instance, if we want to assess the parameter named re10.K1 and this reaction is involved in the Th1 phenotype we want to use the value that has the highest/lowest gradient in our results. In this case, it would coincide with the Th1 parameter estimation. An example is shown in Table S2. As an example, the parameter K1 in reaction number 10 has the lowest gradient in the results of the Th1/Th2 cross-talk parameter estimation. So when uploading these numbers to the model, re10.K1 will have a value of 64.1808, which is the one obtained from the task.

The results on the parameter estimation using Particle Swarm shows a good fitting between the experimental data and the values computationally estimated by COPASI with reduced weighted error (Table S3 and Figure S4). These values are then implemented in the reactions and rate laws to adjust the dynamics of the model, based on the model assumptions considered for the CD4+ T cell model (Table S4).

Once this step is completed, quality control is performed. Using the proper initialization given by literature and represented in Table S5, the system is induced to the four phenotypes and checked to reproduce the correct up- and downregulation of specific molecules.

These four phenotype checks are the result of our CD4+ T cell modeling efforts after calibration and they provide evidence that our computational and mathematical model is capable of reproducing the behaviors of the four CD4+ T cell phenotypes in terms of cytokines, inductors and transcription. In addition, we demonstrate that the calibration process has been run successfully and the dynamics of the CD4+ T cell differentiation network model are adjusted to mimic immunological behaviors characteristic of each phenotype (Figure S5).

**References**

1. Weiner HL (2001) Induction and mechanism of action of transforming growth factor-beta-secreting Th3 regulatory cells. Immunol Rev 182: 207-214.

2. Groux H, O'Garra A, Bigler M, Rouleau M, Antonenko S, et al. (1997) A CD4+ T-cell subset inhibits antigen-specific T-cell responses and prevents colitis. Nature 389: 737-742.

3. Veldhoen M, Uyttenhove C, van Snick J, Helmby H, Westendorf A, et al. (2008) Transforming growth factor-beta 'reprograms' the differentiation of T helper 2 cells and promotes an interleukin 9-producing subset. Nat Immunol 9: 1341-1346.

4. Nurieva RI, Chung Y, Hwang D, Yang XO, Kang HS, et al. (2008) Generation of T follicular helper cells is mediated by interleukin-21 but independent of T helper 1, 2, or 17 cell lineages. Immunity 29: 138-149.

5. Vogelzang A, McGuire HM, Yu D, Sprent J, Mackay CR, et al. (2008) A fundamental role for interleukin-21 in the generation of T follicular helper cells. Immunity 29: 127-137.

6. Mendoza L (2006) A network model for the control of the differentiation process in Th cells. Biosystems 84: 101-114.

7. Kohler B (2007) Mathematically modeling dynamics of T cell responses: predictions concerning the generation of memory cells. J Theor Biol 245: 669-676.

8. Zand MS, Briggs BJ, Bose A, Vo T (2004) Discrete event modeling of CD4+ memory T cell generation. J Immunol 173: 3763-3772.

9. Garcia-Martinez K, Leon K (2010) Modeling the role of IL-2 in the interplay between CD4+ helper and regulatory T cells: assessing general dynamical properties. J Theor Biol 262: 720-732.

10. Zhu J, Yamane H, Paul WE (2010) Differentiation of effector CD4 T cell populations (*). Annu Rev Immunol 28: 445-489.

11. Schulz EG, Mariani L, Radbruch A, Hofer T (2009) Sequential polarization and imprinting of type 1 T helper lymphocytes by interferon-gamma and interleukin-12. Immunity 30: 673-683.

12. Hong T, Xing J, Li L, Tyson JJ (2011) A mathematical model for the reciprocal differentiation of T helper 17 cells and induced regulatory T cells. PLoS Comput Biol 7: e1002122.

13. Naldi A, Carneiro J, Chaouiya C, Thieffry D (2010) Diversity and plasticity of Th cell types predicted from regulatory network modelling. PLoS Comput Biol 6: e1000912.

14. Graw F, Weber KS, Allen PM, Perelson AS (2012) Dynamics of CD4+ T Cell Responses against Listeria monocytogenes. J Immunol.

15. Ying H, Yang L, Qiao G, Li Z, Zhang L, et al. (2010) Cutting edge: CTLA-4--B7 interaction suppresses Th17 cell differentiation. J Immunol 185: 1375-1378.

16. Boothby M (2009) The calculus of integrating differentiation: timing control of T-bet. Immunity 30: 666-668.

17. Barbulescu K, Becker C, Schlaak JF, Schmitt E, Meyer zum Buschenfelde KH, et al. (1998) IL-12 and IL-18 differentially regulate the transcriptional activity of the human IFN-gamma promoter in primary CD4+ T lymphocytes. J Immunol 160: 3642-3647.

18. Horvath CM (2004) The Jak-STAT Pathway Stimulated by Interferon {gamma}. Sci STKE 2004: tr8-.

19. Jaruga B, Hong F, Kim W-H, Gao B (2004) IFN-{gamma}/STAT1 acts as a proinflammatory signal in T cell-mediated hepatitis via induction of multiple chemokines and adhesion molecules: a critical role of IRF-1. Am J Physiol Gastrointest Liver Physiol 287: G1044-1052.

20. Krause CD, He W, Kotenko S, Pestka S (2006) Modulation of the activation of Stat1 by the interferon-[gamma] receptor complex. Cell Res 16: 113-123.

21. Afkarian M, Sedy JR, Yang J, Jacobson NG, Cereb N, et al. (2002) T-bet is a STAT1-induced regulator of IL-12R expression in naive CD4+ T cells. Nat Immunol 3: 549-557.

22. Afkarian M, Sedy JR, Yang J, Jacobson NG, Cereb N, et al. (2002) T-bet is a STAT1-induced regulator of IL-12R expression in naive CD4+ T cells. Nat Immunol 3: 549-557.

23. Mullen AC, High FA, Hutchins AS, Lee HW, Villarino AV, et al. (2001) Role of T-bet in Commitment of TH1 Cells Before IL-12-Dependent Selection. Science 292: 1907-1910.

24. Szabo SJ, Kim ST, Costa GL, Zhang X, Fathman CG, et al. (2000) A novel transcription factor, T-bet, directs Th1 lineage commitment. Cell 100: 655-669.

25. Gunther L, Tilo B, Christoph Sr, Claudia Gn, Julia K, et al. (2004) Sustained T-bet expression confers polarized human TH2 cells with TH1-like cytokine production and migratory capacities. The Journal of allergy and clinical immunology 113: 987-994.

26. Losman J, Chen XP, Jiang H, Pan PY, Kashiwada M, et al. (1999) IL-4 signaling is regulated through the recruitment of phosphatases, kinases, and SOCS proteins to the receptor complex. Cold Spring Harb Symp Quant Biol 64: 405-416.

27. Sato T, Saito R, Jinushi T, Tsuji T, Matsuzaki J, et al. (2004) IFN-gamma-induced SOCS-1 regulates STAT6-dependent eotaxin production triggered by IL-4 and TNF-alpha. Biochem Biophys Res Commun 314: 468-475.

28. Cooney RN (2002) Suppressors of cytokine signaling (SOCS): inhibitors of the JAK/STAT pathway. Shock 17: 83-90.

29. Kimura A, Naka T, Nagata S, Kawase I, Kishimoto T (2004) SOCS-1 suppresses TNF-{alpha}-induced apoptosis through the regulation of Jak activation. Int Immunol 16: 991-999.

30. Furuta S, Kagami S-i, Tamachi T, Ikeda K, Fujiwara M, et al. (2008) Overlapping and Distinct Roles of STAT4 and T-bet in the Regulation of T Cell Differentiation and Allergic Airway Inflammation. J Immunol 180: 6656-6662.

31. Jacobson N, Szabo S, Weber-Nordt R, Zhong Z, Schreiber R, et al. (1995) Interleukin 12 signaling in T helper type 1 (Th1) cells involves tyrosine phosphorylation of signal transducer and activator of transcription (Stat)3 and Stat4. J Exp Med 181: 1755-1762.

32. Park W-R, Nakahira M, Sugimoto N, Bian Y, Yashiro-Ohtani Y, et al. (2004) A mechanism underlying STAT4-mediated up-regulation of IFN-{gamma} induction inTCR-triggered T cells. Int Immunol 16: 295-302.

33. Okamura H, Kashiwamura S-i, Tsutsui H, Yoshimoto T, Nakanishi K (1998) Regulation of interferon-[gamma] production by IL-12 and IL-18. Current Opinion in Immunology 10: 259-264.

34. Nakahira M, Ahn H-J, Park W-R, Gao P, Tomura M, et al. (2002) Synergy of IL-12 and IL-18 for IFN-{gamma} Gene Expression: IL-12-Induced STAT4 Contributes to IFN-{gamma} Promoter Activation by Up-Regulating the Binding Activity of IL-18-Induced Activator Protein 1. J Immunol 168: 1146-1153.

35. Robinson D, Shibuya K, Mui A, Zonin F, Murphy E, et al. (1997) IGIF Does Not Drive Th1 Development but Synergizes with IL-12 for Interferon-[gamma] Production and Activates IRAK and NF[kappa]B. Immunity 7: 571-581.

36. Matsumoto S, Tsuji-Takayama K, Aizawa Y, Koide K, Takeuchi M, et al. (1997) Interleukin-18 Activates NF-[kappa]B in Murine T Helper Type 1 Cells. Biochemical and Biophysical Research Communications 234: 454-457.

37. Akira S (2000) The role of IL-18 in innate immunity. Current Opinion in Immunology 12: 59-63.

38. Yoshimoto T, Takeda K, Tanaka T, Ohkusu K, Kashiwamura S-i, et al. (1998) IL-12 Up-Regulates IL-18 Receptor Expression on T Cells, Th1 Cells, and B Cells: Synergism with IL-18 for IFN-{gamma} Production. J Immunol 161: 3400-3407.

39. Tamachi T, Takatori H, Fujiwara M, Hirose K, Maezawa Y, et al. (2009) STAT6 inhibits T-bet-independent Th1 cell differentiation. Biochemical and Biophysical Research Communications 382: 751-755.

40. Smeltz RB, Chen J, Hu-Li J, Shevach EM (2001) Regulation of Interleukin (IL)-18 Receptor {alpha} Chain Expression on CD4+ T Cells during T Helper (Th)1/Th2 Differentiation: Critical Downregulatory Role of IL-4. J Exp Med 194: 143-154.

41. Yang XO, Panopoulos AD, Nurieva R, Chang SH, Wang D, et al. (2007) STAT3 Regulates Cytokine-mediated Generation of Inflammatory Helper T Cells. J Biol Chem 282: 9358-9363.

42. Bettelli E, Dastrange M, Oukka M (2005) Foxp3 interacts with nuclear factor of activated T cells and NF-ÎºB to repress cytokine gene expression and effector functions of T helper cells. Proceedings of the National Academy of Sciences of the United States of America 102: 5138-5143.

43. Cunard R, Eto Y, Muljadi JT, Glass CK, Kelly CJ, et al. (2004) Repression of IFN-{gamma} Expression by Peroxisome Proliferator-Activated Receptor {gamma}. J Immunol 172: 7530-7536.

44. Wang LH, Yang XY, Zhang X, Huang J, Hou J, et al. (2004) Transcriptional Inactivation of STAT3 by PPAR³ Suppresses IL-6-Responsive Multiple Myeloma Cells. 20: 205-218.

45. Ricote M, Li AC, Willson TM, Kelly CJ, Glass CK (1998) The peroxisome proliferator-activated receptor-gamma is a negative regulator of macrophage activation. Nature 391: 79-82.

46. Kelly D, Campbell JI, King TP, Grant G, Jansson EA, et al. (2004) Commensal anaerobic gut bacteria attenuate inflammation by regulating nuclear-cytoplasmic shuttling of PPAR-gamma and RelA. Nat Immunol 5: 104-112.

47. Zhu J, Yamane H, Cote-Sierra J, Guo L, Paul WE (2006) GATA-3 promotes Th2 responses through three different mechanisms: induction of Th2 cytokine production, selective growth of Th2 cells and inhibition of Th1 cell-specific factors. Cell Res 16: 3-10.

48. Chen Z, Lund R, Aittokallio T, Kosonen M, Nevalainen O, et al. (2003) Identification of Novel IL-4/Stat6-Regulated Genes in T Lymphocytes. J Immunol 171: 3627-3635.

49. Ouyang W, Löhning M, Gao Z, Assenmacher M, Ranganath S, et al. (2000) Stat6-Independent GATA-3 Autoactivation Directs IL-4-Independent Th2 Development and Commitment. 12: 27-37.

50. Cote-Sierra J, Foucras G, Guo L, Chiodetti L, Young HA, et al. (2004) Interleukin 2 plays a central role in Th2 differentiation. Proceedings of the National Academy of Sciences of the United States of America 101: 3880-3885.

51. Zhu J, Cote-Sierra J, Guo L, Paul WE (2003) Stat5 Activation Plays a Critical Role in Th2 Differentiation. 19: 739-748.

52. Yamane H, Zhu J, Paul WE (2005) Independent roles for IL-2 and GATA-3 in stimulating naive CD4+ T cells to generate a Th2-inducing cytokine environment. J Exp Med 202: 793-804.

53. Amsen D, Antov A, Jankovic D, Sher A, Radtke F, et al. (2007) Direct Regulation of Gata3 Expression Determines the T Helper Differentiation Potential of Notch. 27: 89-99.

54. Fang TC, Yashiro-Ohtani Y, Del Bianco C, Knoblock DM, Blacklow SC, et al. (2007) Notch Directly Regulates Gata3 Expression during T Helper 2 Cell Differentiation. Immunity 27: 100-110.

55. Cunard R, Ricote M, DiCampli D, Archer DC, Kahn DA, et al. (2002) Regulation of Cytokine Expression by Ligands of Peroxisome Proliferator Activated Receptors. J Immunol 168: 2795-2802.

56. Yang L, Anderson DE, Baecher-Allan C, Hastings WD, Bettelli E, et al. (2008) IL-21 and TGF-[bgr] are required for differentiation of human TH17 cells. Nature 454: 350-352.

57. Chung SW, Kang BY, Kim TS (2003) Inhibition of interleukin-4 production in CD4+ T cells by peroxisome proliferator-activated receptor-gamma (PPAR-gamma) ligands: involvement of physical association between PPAR-gamma and the nuclear factor of activated T cells transcription factor. Mol Pharmacol 64: 1169-1179.

58. Yang XY, Wang LH, Mihalic K, Xiao W, Chen T, et al. (2002) Interleukin (IL)-4 indirectly suppresses IL-2 production by human T lymphocytes via peroxisome proliferator-activated receptor gamma activated by macrophage-derived 12/15-lipoxygenase ligands. J Biol Chem 277: 3973-3978.

59. Huang JT, Welch JS, Ricote M, Binder CJ, Willson TM, et al. (1999) Interleukin-4-dependent production of PPAR-gamma ligands in macrophages by 12/15-lipoxygenase. Nature 400: 378-382.

60. Yu C-R, Mahdi RM, Ebong S, Vistica BP, Chen J, et al. (2004) Cell Proliferation and STAT6 Pathways Are Negatively Regulated in T Cells by STAT1 and Suppressors of Cytokine Signaling. J Immunol 173: 737-746.

61. Hebenstreit D, Luft P, Schmiedlechner A, Duschl A, Horejs-Hoeck J (2005) SOCS-1 and SOCS-3 inhibit IL-4 and IL-13 induced activation of Eotaxin-3/CCL26 gene expression in HEK293 cells. Molecular Immunology 42: 295-303.

62. Losman JA, Chen XP, Hilton D, Rothman P (1999) Cutting Edge: SOCS-1 Is a Potent Inhibitor of IL-4 Signal Transduction. J Immunol 162: 3770-3774.

63. Venkataraman C, Leung S, Salvekar A, Mano H, Schindler U (1999) Repression of IL-4-Induced Gene Expression by IFN-{gamma} Requires Stat1 Activation. J Immunol 162: 4053-4061.

64. Huang Z, Xin J, Coleman J, Huang H (2005) IFN-{gamma} Suppresses STAT6 Phosphorylation by Inhibiting Its Recruitment to the IL-4 Receptor. J Immunol 174: 1332-1337.

65. Gorelik L, Fields PE, Flavell RA (2000) Cutting Edge: TGF-{beta} Inhibits Th Type 2 Development Through Inhibition of GATA-3 Expression. J Immunol 165: 4773-4777.

66. Torgerson TR, Genin A, Chen C, Zhang M, Zhou B, et al. (2009) FOXP3 Inhibits Activation-Induced NFAT2 Expression in T Cells Thereby Limiting Effector Cytokine Expression. J Immunol 183: 907-915.

67. Bettelli E, Korn T, Kuchroo VK (2007) Th17: the third member of the effector T cell trilogy. Current Opinion in Immunology 19: 652-657.

68. Zhou L, Lopes JE, Chong MMW, Ivanov II, Min R, et al. (2008) TGF-[bgr]-induced Foxp3 inhibits TH17 cell differentiation by antagonizing ROR[ggr]t function. Nature 453: 236-240.

69. Bettelli E, Carrier Y, Gao W, Korn T, Strom TB, et al. (2006) Reciprocal developmental pathways for the generation of pathogenic effector TH17 and regulatory T cells. Nature 441: 235-238.

70. Korn T, Bettelli E, Gao W, Awasthi A, Jager A, et al. (2007) IL-21 initiates an alternative pathway to induce proinflammatory TH17 cells. Nature 448: 484-487.

71. Zhou L, Ivanov II, Spolski R, Min R, Shenderov K, et al. (2007) IL-6 Programs TH-17 Cell Differentiation by Promoting the Sequential Engagement of the IL-21 and IL-23 Pathways. Cytokine 39: 49-49.

72. Foley JF (2007) STAT3 Regulates the Generation of Th17 Cells. Sci STKE 2007: tw113-.

73. Harris TJ, Grosso JF, Yen H-R, Xin H, Kortylewski M, et al. (2007) Cutting Edge: An In Vivo Requirement for STAT3 Signaling in TH17 Development and TH17-Dependent Autoimmunity. J Immunol 179: 4313-4317.

74. Mathur AN, Chang H-C, Zisoulis DG, Stritesky GL, Yu Q, et al. (2007) Stat3 and Stat4 Direct Development of IL-17-Secreting Th Cells. J Immunol 178: 4901-4907.

75. Ivanov II, McKenzie BS, Zhou L, Tadokoro CE, Lepelley A, et al. (2006) The Orphan Nuclear Receptor ROR³t Directs the Differentiation Program of Proinflammatory IL-17+ T Helper Cells. 126: 1121-1133.

76. McGeachy MJ, Chen Y, Tato CM, Laurence A, Joyce-Shaikh B, et al. (2009) The interleukin 23 receptor is essential for the terminal differentiation of interleukin 17-producing effector T helper cells in vivo. Nat Immunol 10: 314-324.

77. Korn T, Bettelli E, Oukka M, Kuchroo VK (2009) IL-17 and Th17 Cells. Annual Review of Immunology 27: 485-517.

78. Laurence A, O'Shea JJ (2007) TH-17 differentiation: of mice and men. Nat Immunol 8: 903-905.

79. Volpe E, Servant N, Zollinger R, Bogiatzi SI, Hupe P, et al. (2008) A critical function for transforming growth factor-[beta], interleukin 23 and proinflammatory cytokines in driving and modulating human TH-17 responses. Nat Immunol 9: 650-657.

80. Awasthi A, Murugaiyan G, Kuchroo V (2008) Interplay Between Effector Th17 and Regulatory T Cells. Journal of Clinical Immunology 28: 660-670.

81. Mathur AN, Chang HC, Zisoulis DG, Kapur R, Belladonna ML, et al. (2006) T-bet is a critical determinant in the instability of the IL-17-secreting T-helper phenotype. Blood 108: 1595-1601.

82. Gocke AR, Cravens PD, Ben LH, Hussain RZ, Northrop SC, et al. (2007) T-bet regulates the fate of Th1 and Th17 lymphocytes in autoimmunity. J Immunol 178: 1341-1348.

83. van Hamburg JP, de Bruijn MJ, Ribeiro de Almeida C, van Zwam M, van Meurs M, et al. (2008) Enforced expression of GATA3 allows differentiation of IL-17-producing cells, but constrains Th17-mediated pathology. Eur J Immunol 38: 2573-2586.

84. Ichiyama K, Yoshida H, Wakabayashi Y, Chinen T, Saeki K, et al. (2008) Foxp3 Inhibits ROR{gamma}t-mediated IL-17A mRNA Transcription through Direct Interaction with ROR{gamma}t. J Biol Chem 283: 17003-17008.

85. Passerini L, Allan SE, Battaglia M, Di Nunzio S, Alstad AN, et al. (2008) STAT5-signaling cytokines regulate the expression of FOXP3 in CD4+CD25+ regulatory T cells and CD4+CD25- effector T cells. Int Immunol 20: 421-431.

86. Wei L, Laurence A, Elias KM, O'Shea JJ (2007) IL-21 is produced by Th17 cells and drives IL-17 production in a STAT3-dependent manner. J Biol Chem 282: 34605-34610.

87. Tartar DM, VanMorlan AM, Wan X, Guloglu FB, Jain R, et al. (2010) FoxP3+RORgammat+ T helper intermediates display suppressive function against autoimmune diabetes. J Immunol 184: 3377-3385.

88. Lochner M, Peduto L, Cherrier M, Sawa S, Langa F, et al. (2008) In vivo equilibrium of proinflammatory IL-17+ and regulatory IL-10+ Foxp3+ RORgamma t+ T cells. J Exp Med 205: 1381-1393.

89. Laurence A, Tato CM, Davidson TS, Kanno Y, Chen Z, et al. (2007) Interleukin-2 signaling via STAT5 constrains T helper 17 cell generation. Immunity 26: 371-381.

90. Chen Y, Haines CJ, Gutcher I, Hochweller K, Blumenschein WM, et al. (2011) Foxp3(+) regulatory T cells promote T helper 17 cell development in vivo through regulation of interleukin-2. Immunity 34: 409-421.

91. Kimura A, Naka T, Nohara K, Fujii-Kuriyama Y, Kishimoto T (2008) Aryl hydrocarbon receptor regulates Stat1 activation and participates in the development of Th17 cells. Proceedings of the National Academy of Sciences 105: 9721-9726.

92. Li B, Reynolds JM, Stout RD, Bernlohr DA, Suttles J (2009) Regulation of Th17 Differentiation by Epidermal Fatty Acid-Binding Protein. J Immunol 182: 7625-7633.

93. Klotz L, Burgdorf S, Dani I, Saijo K, Flossdorf J, et al. (2009) The nuclear receptor PPAR gamma selectively inhibits Th17 differentiation in a T cell-intrinsic fashion and suppresses CNS autoimmunity. J Exp Med 206: 2079-2089.

94. Pyzik M, Piccirillo CA (2007) TGF-beta1 modulates Foxp3 expression and regulatory activity in distinct CD4+ T cell subsets. J Leukoc Biol 82: 335-346.

95. Chen W, Jin W, Hardegen N, Lei K-j, Li L, et al. (2003) Conversion of Peripheral CD4+CD25- Naive T Cells to CD4+CD25+ Regulatory T Cells by TGF-{beta} Induction of Transcription Factor Foxp3. J Exp Med 198: 1875-1886.

96. Pyzik M, Piccirillo CA (2007) TGF-{beta}1 modulates Foxp3 expression and regulatory activity in distinct CD4+ T cell subsets. J Leukoc Biol 82: 335-346.

97. Chen W, Jin W, Hardegen N, Lei KJ, Li L, et al. (2003) Conversion of peripheral CD4+CD25- naive T cells to CD4+CD25+ regulatory T cells by TGF-beta induction of transcription factor Foxp3. J Exp Med 198: 1875-1886.

98. Murawski MR, Litherland SA, Clare-Salzler MJ, Davoodi-Semiromi A (2006) Upregulation of Foxp3 expression in mouse and human Treg is IL-2/STAT5 dependent: implications for the NOD STAT5B mutation in diabetes pathogenesis. Ann N Y Acad Sci 1079: 198-204.

99. Kitani A, Xu L (2008) Regulatory T cells and the induction of IL-17. Mucosal Immunol 1 Suppl 1: S43-46.

100. Nishibori T, Tanabe Y, Su L, David M (2004) Impaired Development of CD4+ CD25+ Regulatory T Cells in the Absence of STAT1: Increased Susceptibility to Autoimmune Disease. J Exp Med 199: 25-34.

101. Wang Z (2006) Role of IFN-Î³ in induction of Foxp3 and conversion of CD4+ CD25â€“ T cells to CD4+ Tregs. The Journal of Clinical Investigation 116: 2434-2441.

102. Ouaked N, Mantel P-Y, Bassin C, Burgler S, Siegmund K, et al. (2009) Regulation of the foxp3 Gene by the Th1 Cytokines: The Role of IL-27-Induced STAT1. J Immunol 182: 1041-1049.

103. Housley WJ, O'Conor CA, Nichols F, Puddington L, Lingenheld EG, et al. (2009) PPAR{gamma} regulates retinoic acid-mediated DC induction of Tregs. J Leukoc Biol: jlb.1208733.

104. Wohlfert EA, Nichols FC, Nevius E, Clark RB (2007) Peroxisome Proliferator-Activated Receptor {gamma} (PPAR{gamma}) and Immunoregulation: Enhancement of Regulatory T Cells through PPAR{gamma}-Dependent and -Independent Mechanisms. J Immunol 178: 4129-4135.

105. Korn T, Mitsdoerffer M, Croxford AL, Awasthi A, Dardalhon VrA, et al. (2008) IL-6 controls Th17 immunity in vivo by inhibiting the conversion of conventional T cells into Foxp3+ regulatory T cells. Proceedings of the National Academy of Sciences 105: 18460-18465.

106. Mantel P-Y, Kuipers H, Boyman O, Rhyner C, Ouaked N, et al. (2007) GATA3-Driven Th2 Responses Inhibit TGF-Î²1â€“Induced FOXP3 Expression and the Formation of Regulatory T Cells. PLoS Biol 5: e329.

107. Dardalhon V, Awasthi A, Kwon H, Galileos G, Gao W, et al. (2008) IL-4 inhibits TGF-beta-induced Foxp3+ T cells and, together with TGF-beta, generates IL-9+ IL-10+ Foxp3(-) effector T cells. Nat Immunol 9: 1347-1355.

108. Yao Z, Kanno Y, Kerenyi M, Stephens G, Durant L, et al. (2007) Nonredundant roles for Stat5a/b in directly regulating Foxp3. Blood 109: 4368-4375.

109. Chang JH, Kim YJ, Han SH, Kang CY (2009) IFN-gamma-STAT1 signal regulates the differentiation of inducible Treg: potential role for ROS-mediated apoptosis. Eur J Immunol 39: 1241-1251.

110. Chang J-H, Kang C-Y (2008) Autocrine IFN-gamma directly regulates Foxp3 expression in naive CD4+CD25- T cells. FASEB J 22: 848.816-.

111. Koch MA, Tucker-Heard G, Perdue NR, Killebrew JR, Urdahl KB, et al. (2009) The transcription factor T-bet controls regulatory T cell homeostasis and function during type 1 inflammation. Nat Immunol 10: 595-602.

112. Yang XO, Nurieva R, Martinez GJ, Kang HS, Chung Y, et al. (2008) Molecular antagonism and plasticity of regulatory and inflammatory T cell programs. Immunity 29: 44-56.

113. Zhou L, Lopes JE, Chong MM, Ivanov, II, Min R, et al. (2008) TGF-beta-induced Foxp3 inhibits T(H)17 cell differentiation by antagonizing RORgammat function. Nature 453: 236-240.

114. Carter AB, Misyak SA, Hontecillas R, Bassaganya-Riera J (2009) Dietary Modulation of Inflammation-Induced Colorectal Cancer through PPARgamma. PPAR Res 2009: 498-352.

115. Evans NP, Misyak SA, Schmelz EM, Guri AJ, Hontecillas R, et al. (2010) Conjugated linoleic acid ameliorates inflammation-induced colorectal cancer in mice through PPAR gamma. J Nutr In Press.

116. Guri AJ, Hontecillas R, Bassaganya-Riera J (2006) Peroxisome proliferator-activated receptors: Bridging metabolic syndrome with molecular nutrition. Clin Nutr 25: 871-885.

117. Guri AJ, Hontecillas R, Ferrer G, Casagran O, Wankhade U, et al. (2008) Loss of PPAR gamma in immune cells impairs the ability of abscisic acid to improve insulin sensitivity by suppressing monocyte chemoattractant protein-1 expression and macrophage infiltration into white adipose tissue. J Nutr Biochem 19: 216-228.

118. Guri AJ, Hontecillas R, Si H, Liu D, Bassaganya-Riera J (2007) Dietary abscisic acid ameliorates glucose tolerance and obesity-related inflammation in db/db mice fed high-fat diets. Clin Nutr 26: 107-116.

119. Guri AJ, Misyak SA, Hontecillas R, Hasty A, Liu D, et al. (2010) Abscisic acid ameliorates atherosclerosis by suppressing macrophage and CD4+ T cell recruitment into the aortic wall. J Nutr Biochem 21: 1178-1185.

120. Bassaganya-Riera J, Misyak S, Guri AJ, Hontecillas R (2009) PPAR gamma is highly expressed in F4/80(hi) adipose tissue macrophages and dampens adipose-tissue inflammation. Cell Immunol 258: 138-146.

121. Bassaganya-Riera J, Hontecillas R, Zimmerman DR, Wannemuehler MJ (2001) Dietary conjugated linoleic acid modulates phenotype and effector functions of porcine cd8(+) lymphocytes. J Nutr 131: 2370-2377.

122. Bassaganya-Riera J, Pogranichniy RM, Jobgen SC, Halbur PG, Yoon KJ, et al. (2003) Conjugated linoleic acid ameliorates viral infectivity in a pig model of virally induced immunosuppression. J Nutr 133: 3204-3214.

123. Paranavitana C, Pittman PR, Velauthapillai M, Zelazowska E, Dasilva L (2008) Transcriptional profiling of Francisella tularensis infected peripheral blood mononuclear cells: a predictive tool for tularemia. FEMS Immunol Med Microbiol 54: 92-103.

124. Aldridge JR, Jr., Moseley CE, Boltz DA, Negovetich NJ, Reynolds C, et al. (2009) TNF/iNOS-producing dendritic cells are the necessary evil of lethal influenza virus infection. Proc Natl Acad Sci U S A 106: 5306-5311.

125. Mohapatra SK, Cole LE, Vogel SN, Evans C, Sobral BW, et al. (2010) Murine hepatic gene expression changes during LPS-induced protection of host from infection with Francisella tularensis LVS. BMC Infectious Diseases In Press.

126. Hontecillas R, Wannemeulher MJ, Zimmerman DR, Hutto DL, Wilson JH, et al. (2002) Nutritional regulation of porcine bacterial-induced colitis by conjugated linoleic acid. J Nutr 132: 2019-2027.

127. Bassaganya-Riera J, Reynolds K, Martino-Catt S, Cui Y, Hennighausen L, et al. (2004) Activation of PPAR gamma and delta by conjugated linoleic acid mediates protection from experimental inflammatory bowel disease. Gastroenterology 127: 777-791.

128. Bassaganya-Riera J, Hontecillas R (2006) CLA and n-3 PUFA differentially modulate clinical activity and colonic PPAR-responsive gene expression in a pig model of experimental IBD. Clin Nutr 25: 454-465.

129. Hontecillas R, Bassaganya-Riera J (2007) Peroxisome proliferator-activated receptor gamma is required for regulatory CD4+ T cell-mediated protection against colitis. J Immunol 178: 2940-2949.

130. Wohlfert E, Clark RB. A novel approach for modulating CD4+CD25+ regulatory T cell generation and function through the nuclear receptor PPAR g; 2006; Boston, MA. J. Immunol. pp. S144.

131. Klotz L, Burgdorf S, Dani I, Saijo K, Flossdorf J, et al. (2009) The nuclear receptor PPAR gamma selectively inhibits Th17 differentiation in a T cell-intrinsic fashion and suppresses CNS autoimmunity. J Exp Med 206: 2079-2089.

132. Hevener AL, Olefsky JM, Reichart D, Nguyen MT, Bandyopadyhay G, et al. (2007) Macrophage PPAR gamma is required for normal skeletal muscle and hepatic insulin sensitivity and full antidiabetic effects of thiazolidinediones. J Clin Invest 117: 1658-1669.

133. Wendelsdorf K, Bassaganya-Riera J, Hontecillas R, Eubank S (2010) Model of colonic inflammation: immune modulatory mechanisms in inflammatory bowel disease. J Theor Biol 264: 1225-1239.

134. Funahashi A MM, Tanimura N, Kitano H (2003) CellDesigner: a process diagram editor for gene-regulatory and biochemical networks. BioSilico 1: 159-162.

135. Hoops S, Sahle S, Gauges R, Lee C, Pahle J, et al. (2006) COPASI--a COmplex PAthway SImulator. Bioinformatics 22: 3067-3074.

136. Kennedy JE, R. (1995) Particle Swarm Optimization. Neural Networks, IEEE International Conference 4: 1942-1948.

137. Xu R, Venayagamoorthy GK, Wunsch DC, 2nd (2007) Modeling of gene regulatory networks with hybrid differential evolution and particle swarm optimization. Neural Netw 20: 917-927.
